# Characterizing resistant cellular states in nasopharyngeal carcinoma during EBV lytic induction

**DOI:** 10.1038/s41388-025-03341-z

**Published:** 2025-03-25

**Authors:** Xinlei Wang, Lei Yu, Xuemeng Zhou, Grace Tin-Yun Chung, Alyssa Ming-Ting Liu, Yuk-Yu Chan, Man Wu, Kin Yung Chau, Kwok-Wai Lo, Angela Ruohao Wu

**Affiliations:** 1 Division of Life Science, The Hong Kong University of Science and Technology, Hong Kong SAR, China; 2 Department of Anatomical and Cellular Pathology, Prince of Wales Hospital, The Chinese University of Hong Kong, Hong Kong SAR, China; 3 Department of Chemical and Biological Engineering, The Hong Kong University of Science and Technology, Hong Kong SAR, China; 4 State Key Laboratory of Molecular Neuroscience, The Hong Kong University of Science and Technology, Hong Kong SAR, China; 5 Center for Aging Science, The Hong Kong University of Science and Technology, Hong Kong SAR, China

**Keywords:** Head and neck cancer, Cancer stem cells

## Abstract

The pervasive occurrence of nasopharyngeal carcinoma (NPC) is intricately linked to Epstein–Barr virus (EBV) infection, making EBV and its associated pathways promising therapeutic targets for NPC and other EBV-related cancers. Lytic induction therapy, an emerging virus-targeted therapeutic strategy, capitalizes on the presence of EBV in tumor cells to specifically induce cytotoxicity against EBV-associated malignancies. Despite the expanding repertoire of compounds developed to induce EBV lytic reactivation, achieving universal induction across all infected cells remains elusive. The inherent heterogeneity of tumor cells likely contributes to this variability. In this study, we used the NPC43 cell line, an EBV-positive NPC in vitro model, and single-cell transcriptomics to characterize the diverse cellular responses to EBV lytic induction. Our longitudinal monitoring revealed a distinctive lytic induction non-responsive cellular state characterized by elevated expression of *SOX2* and *NTRK2*. Cells in this state exhibit phenotypic similarities to cancer stem cells (CSCs), and we verified the roles of *SOX2* and *NTRK2* in manifesting these phenotypes. Our findings reveal a significant challenge for lytic induction therapy, as not all tumor cells are equally susceptible. These insights highlight the importance of combining lytic induction with therapies targeting CSC-like properties to enhance treatment efficacy for NPC and other EBV-associated cancers.

## Introduction

Nasopharyngeal carcinoma (NPC) originates in the nasopharynx and exhibits a distinct geographical distribution [1]. The World Health Organization (WHO) classifies NPC into three subtypes based on its histological characteristics: Differentiated tumors with surface keratin are defined as type I, whereas types II and III refer to non-keratinizing differentiated and undifferentiated tumors [2–5]. Among these subtypes, type III is the most prevalent in regions with a high incidence of NPC, such as southern China and Southeast Asia, and it is invariably associated with Epstein–Barr virus (EBV) infection [6, 7].

The role of EBV in tumorigenesis of non-keratinizing NPC has been extensively explored [8]. Two non-synonymous variants in the EBV genome, specifically in *BALF2*, are linked to a high risk of NPC in southern China, accounting for up to 83% of the total risk in this region [9]. While the precise mechanism underlying the transition from normal epithelial cells to NPC tumors remains unclear, existing models offer insights into the key pathways involved in this progression [10]. To comprehensively understand NPC tumors, in vitro NPC models with endogenous EBV infection are crucial for replicating in vivo tumor features. The newly established NPC cell line, NPC43, is notable for harboring endogenous EBV infection and producing infectious EBV particles following specific lytic induction treatments [11]. The capacity to sustain EBV episomes distinguishes NPC43 cells from other EBV-negative NPC cell lines. Although C666-1 and C17 are EBV-positive NPC cell lines, they were derived from NPC xenografts (xeno-X666 and xeno-C17). C666-1 exhibits defects in undergoing productive lytic EBV infection in response to drug treatments or ectopic overexpression of the *BZLF1* gene [11, 12]. C17 contains only 2–3 copies of the EBV genome per cell and is resistant to all known lytic-inducing drugs [13]. Furthermore, since NPC tumors are typically small and contain a limited proportion of malignant cells, obtaining sufficient samples for extensive analyses can be challenging. EBV-positive NPC43 can provide sufficient experimental materials and better scalability for understanding and optimizing EBV activation [14–18]. The removal of Y-27632, a ROCK inhibitor, induces EBV activation in NPC43, with approximately 10% expression of the immediate-early protein Zta [11]. Furthermore, withdrawing Y-27632 and adding TPA significantly activated EBV in NPC43. Thus, the NPC43 cell line is an emerging model system that is useful and effective for studying NPC biology and, to some extent, its response to therapy.

Given the high prevalence of EBV in NPC, the virus has been proposed as a potential therapeutic target [19]. Lytic induction therapy represents a virus-targeted approach that leverages the presence of EBV in tumor cells to induce specific killing effects against EBV-associated malignancies. This therapeutic strategy involves two key components: lytic inducers and nucleoside analog antivirals (e.g., ganciclovir). The success of the therapy relies significantly on the efficiency of lytic inducers in reactivating the EBV lytic cycle. Therefore, understanding the heterogeneous response of NPC cells to lytic induction is crucial for increasing the efficiency of EBV lytic reactivation in these cells. Given the pronounced heterogeneity of tumor cells, it is plausible that malignant cells may exhibit diverse responses to lytic induction treatment. Consequently, high-resolution characterization of the lytic induction process using single-cell RNA sequencing (scRNA-seq) could be instrumental in capturing and reflecting the intrinsic heterogeneity of NPC cells.

The heterogeneity observed in NPC cells can potentially affect the efficiency of lytic induction. Consequently, gaining insights into the classification of NPC cells becomes imperative. Cancer stem cells (CSCs) or tumor-initiating cells (TICs) have been recognized as a pivotal cellular state in cancer [20–22]. Therefore, investigating the interplay between CSCs and EBV stands out as an essential area of study. In NPC, previous studies identified a subset of cells within the C666-1 cell line with enhanced sphere-forming ability, designating them as CSCs due to heightened tumor formation and chemoresistance capacities [23]. Notably, specific markers of CSCs, such as *SOX2* and *CD44*, have been detected in these specific cells [23]. The existence of CSCs populations and their critical roles in tumor growth, chemoresistance capacity, and ability to repopulate the tumor have been reported in other cancers, emphasizing the significance of identifying and understanding such populations [20–22]. Although *SOX2* traditionally serves as a marker for maintaining stemness, its role in oncogenesis remains debated across different cancers [23–26]. Thus, identifying cell markers specific to CSCs in NPC is imperative for a complete understanding of this cancer, as well as for identifying effective therapies.

In this study, we used NPC cell lines with endogenous EBV infection to assess the feasibility of lytic induction therapy in NPC. First, we used scRNA-seq to analyze the heterogeneous response of NPC43 cells to TPA-based lytic induction. Within this dataset, we identified a subset of cells resistant to EBV lytic induction treatment, termed non-responsive (NR) cells. These NR cells highly expressed *NTRK2* and *SOX2*, exhibiting properties reminiscent of CSCs. Next, we validated the critical roles of *SOX2* and *NTRK2* as key regulators in maintaining the properties of NR cells through spheroid formation and migration assays. To generalize these findings in NPC, we conducted a meta-correlation analysis, which revealed a robust association between *NTRK2* and stemness features across multiple patient tumor datasets [14–18, 27]. Additionally, we further validated the characteristics of *NTRK2*-high/*SOX2*-high cells in the C666-1 cell line by conducting lytic induction treatments using Sodium Butyrate (NaB), followed by scRNA-seq analysis. Consistently, this subpopulation exhibited molecular features associated with stemness and significantly lower expression of EBV genes.

## Methods

### Cell culture and treatments

NPC43, established from nasopharyngeal carcinoma (NPC) tissue of a 64-year-old male patient, and C17, derived from patient-derived xenografts (C17 xenograft was established from NPC tissue of a 39-year-old male patient), were cultured in RPMI 1640 medium (Gibco 23400021) supplemented with 10% fetal bovine serum (FBS) (Gibco 10270106) and 1% penicillin-streptomycin (10,000 U/mL) (Gibco 15140122) in the presence of 4 μM Rho kinase inhibitor Y-27632 dihydrochloride (Abcam 120129). C666-1 cells, derived from patient-derived xenografts (Xeno-666, established from the NPC tissue of a male patient), were maintained in RPMI 1640 medium (Gibco 23400021) supplemented with 10% FBS (Gibco 10270106). NP69 and NP460 cells were cultured in a mixture of EpiLife Medium (Gibco MEPI500CA) with 60 μM calcium and Defined Keratinocyte SFM (1×) (Gibco 10744019) in a 1:1 ratio, supplemented with 10% FBS (Gibco 10270106) and 1% penicillin-streptomycin (10,000 U/mL) (Gibco 15140122). All cells were cultured in T75 flasks (Nunc 156800). When reaching 75–90% confluence, they were detached using a 0.05% trypsin/EDTA solution (Gibco 25300062). The cells were maintained at 37 °C with 5% CO_2_ in humidified air.

To induce the lytic cycle of Epstein–Barr virus (EBV) in NPC43, we replaced the RPMI 1640 medium without Y-27632 and supplemented it with 40 ng/ml tetradecanoyl phorbol acetate (TPA) (Sigma–Aldrich, P8139). After the induction treatment, cells were maintained in the same medium for 24 or 48 h. To induce lytic activation in C666-1 cells, the cells were cultured in fresh RPMI 1640 medium supplemented with 3 mM NaB (Sigma–Aldrich, B5887). All cell lines were tested for mycoplasma contamination by polymerase chain reaction (PCR).

### Single-cell RNA-seq

Cell lines were cultured and subjected to lytic induction for 24 or 48 h. Two biological replicates were used for untreated cells and 48-h treated cells, while one replicate was used for 24-h treated cells. Cells were harvested from T75 flasks using Trypsin-EDTA (0.05%) and washed twice with 1× phosphate-buffered saline (PBS) (Gibco, #10010049). Cells were resuspended in ACCUMAX (STEMCELL 07921) or PBS-T-BSA buffer (1 mM EDTA, 0.3% BSA), depending on their tendency to aggregate, and filtered using a Falcon cell strainer (Falcon, #100-0087). Cell viability was assessed using an automated cell counter with trypan blue staining. Subsequently, cells were diluted to a concentration of 5 × 10⁵ cells/ml, and dead cells were labeled with Propidium Iodide (PI, 3 μM). Single cells were enriched in PBS-T-BSA buffer using the BD Influx cell sorter before being loaded onto the 10X Chromium Controller. To prepare the single-cell suspension for the 10X Chromium platform, cells were concentrated to a final range of 5 × 10⁵ to 10⁶ cells/ml.

The single-cell RNA-seq (scRNA-seq) library was prepared following the Chromium Single &&Cell 3′ Reagent Kits User Guide (V3 Chemistry, V3.1 Chemistry) from 10X GENOMICS. In each run, we attempted to sequence 5000 cells. The concentration of the resulting library was quantified using the Qubit3.0 with the Qubit HS DNA kit (Thermo Fisher Q32854), and the library size was assessed using a DNF-474 High-Sensitivity NGS Fragment Analysis kit (1–6000 bp). Sequencing was performed on an Illumina Nextseq 500/550 sequencer using the NextSeq 500/550 High Output Kit v2.5 (Illumina 20024907, 150 cycles) in paired-end mode.

The raw data were processed using Cell Ranger 4.0.0, which includes demultiplexing of raw base call (BCL) files into FASTQ files, alignment, and Unique Molecular Identifier (UMI) counting. Reads were aligned to the Homo sapiens genome assembly GRCh38 (hg38).

### Single-cell RNA-seq data processing

The Seurat package (version 5) was employed to import the gene matrix into R and conduct subsequent analyses. Initial quality control involved filtering out low-quality cells based on specific criteria: cells were required to have more than 1000 detected genes, with mitochondrial reads comprising less than 25% of the total reads. Additionally, genes detected in fewer than 5 cells were removed. The SCTransform function was applied to normalize the data. Principal Component Analysis (PCA) was performed for dimensionality reduction using default parameters. For visualization purposes, the Uniform Manifold Approximation and Projection (UMAP) technique was employed via the RunUMAP function with 50 Principal Components (PCs).

Cells from the three time points were clustered using the Leiden algorithm, implemented through the FindNeighbors and FindClusters functions. Integration of the datasets from the three time points was achieved using the FindIntegrationAnchors and IntegrateData functions in Seurat. To annotate cellular states, differential gene expression analysis was conducted using the FindAllMarkers function. Clusters with similar gene expression patterns in UMAP were merged accordingly.

To establish corresponding relationships between clusters across the three time points, Spearman's correlation analysis was performed on pseudo-bulks at the cluster level using the top 1000 variable genes from each time point. Clusters that share similar cellular states are expected to exhibit higher levels of similarity. Gene signature sets were obtained from The Molecular Signatures Database (MSigDB), and the UCell method was employed to score the activity level of gene sets. Differential activation of gene sets was identified using the Wilcoxon Rank Sum test.

Cellular states were annotated based on active gene sets and differentially expressed genes (DEGs). Furthermore, to validate the response to TPA treatment and withdrawal of the Rho kinase inhibitor, a Pathway RespOnsive GENes (PROGENy) analysis was conducted. This analysis is based on the footprint of the pathway on gene expression [28].

Cellular state trajectory analyses were performed using PAGA and DPT within the Scanpy framework [29]. To ensure consistency with analyses conducted in Seurat, SCT-normalized data were extracted as the normalized input for Scanpy. UMAP embeddings were recalculated using PAGA, resulting in a visualization that showed significant differences compared to the original UMAP. DPT was subsequently calculated using the sc.tl.dpt function, with the top 10% of NTRK2-high cells designated as the root population. The DPT results were then transformed into velocity-like vectors using CellRank [30].

### Gene network analysis

SCENIC (Single-Cell rEgulatory Network Inference and Clustering) is a computational tool utilized to infer regulatory modules or regulons by analyzing the co-expression of transcription factors (TFs) and their putative target genes, characterized by the enrichment of corresponding transcription factor-binding motifs in their regulatory regions.

In this study, regulatory network analysis was conducted on all NPC43 cells using the Python package pySCENIC with default parameters. Transcription factors potentially regulating *NTRK2* were extracted from the inferred regulons. Subsequently, a network analysis was performed using the R igraph package. The all_simple_paths function was used to identify all related transcription factors between *NTRK2* and *SOX2*.

The resulting network was then imported into Cytoscape for visualization, allowing for a comprehensive and intuitive representation of the regulatory interactions between *NTRK2*, *SOX2*, and their associated transcription factors.

The necessary codes to reproduce the analysis workflow were deposited in GitHub (https://github.com/0YuLei0/NPC43-single-cell-analysis).

### Gene sets analysis

Meta-correlation analysis was performed on both bulk RNA-seq and scRNA-seq data from NPC patients [15–18]. Four gene set databases were utilized in this study. Three of these were curated from established resources, including Gene Ontology (GO), Reactome, and pan-cancer gene sets [31]. The fourth, NPC-specific gene sets, was generated using non-negative matrix factorization (NMF) analysis applied to scRNA-seq data from NPC patients (Supplementary Table S2). The scRNA-seq data used in this analysis can be accessed from the original publication or the Curated Cancer Cell Atlas (https://www.weizmann.ac.il/sites/3CA/). The detailed code for NMF analysis is also available in the 3CA repository.

For bulk RNA-seq datasets, gene set activities were quantified using Gene Set Variation Analysis (GSVA) [32]. For scRNA-seq datasets, gene set activities were calculated using the Ucell algorithm. Pearson correlation analyses were then performed to assess the relationship between NTRK2 expression and other features across different datasets. Genes or gene sets demonstrating consistent correlation with NTRK2 (R > 0.3) across multiple datasets were retained for further analysis.

### FACS analysis and sorting

For live cell staining, NPC43 cells were harvested and washed with phosphate-buffered saline (PBS) prior to staining. The cells were then incubated with staining buffer (0.5% bovine serum albumin (BSA) in PBS) containing human TrkB Alexa Fluor® 488-conjugated Antibody (R&D Systems, FAB3971G) with gentle shaking for 15 min at room temperature. Subsequently, the cells were washed with staining buffer to remove any residual antibody before proceeding to fluorescence-activated cell sorting (FACS).

For intracellular staining, harvested cells were fixed using 0.01% formaldehyde for 10 min at room temperature. After fixation, the cellular membrane was permeabilized using PBS containing 0.25% Triton X-100. The cell suspension was centrifuged at 1000 g for 5 min, and the supernatant was carefully removed. The cell pellet was washed with PBS containing 0.1% Triton X-100 and centrifuged again at 1000 g for 5 min. Finally, the cells were resuspended in PBS containing 3% BSA. Primary antibodies against SOX2 (Recombinant Anti-SOX2 antibody [EPR3131], Abcam, ab92494), NTRK2 (Human TrkB Alexa Fluor 488-conjugated Antibody, R&D Systems, FAB3971G), and ZEBRA Antibody (BZ1) (Santa Cruz Biotechnology, sc-53904) were added at a 1:4000 dilution, and the cells were incubated at 4 °C for 20 min. Following primary antibody incubation, the cells were washed with PBS containing 3% BSA and then stained with fluorescent-dye conjugated secondary antibodies (Goat Anti-Rabbit IgG H&L, Alexa Fluor® 647, Abcam, ab150079) at room temperature for 30 min. After staining, the cells were washed with PBS containing 3% BSA and then subjected to FACS analysis.

Cell sorting was performed using a BD Influx Cell Sorter, and the recorded data were analyzed using the FlowJo v10 software. This approach enabled the identification and isolation of specific cell populations based on their fluorescence profiles, thereby facilitating further downstream analysis and experimentation.

### DNA and RNA extraction, cDNA library construction, and quantitative real-time PCR

Cells from multiple flasks were harvested and lysed in TRIzol (Gibco, 15596018) to facilitate nucleic acid extraction. RNA extraction was performed according to the manufacturer’s protocol, and DNA extraction was performed using the PureLink Genomic DNA Mini Kit (Invitrogen K182001). The concentration of extracted RNA was measured using the Qubit RNA High Sensitivity (HS) Assay Kit (Q32855), and the concentration of DNA was determined using the Qubit HS dsDNA Assay Kit. The cDNA library was constructed using the Smart-seq2 method. The cDNA-PCR product was purified using AMPure XP (BECKMAN A63882), and its concentration was quantified using the Qubit HS dsDNA Assay Kit.

The expression of specific genes was assessed using qPCR with LightCycler SYBR Green I Master (Roche 04887352001). The reaction system was assembled according to the manufacturer’s protocol and run on a Roche LightCycler480 real-time PCR system. Relative gene expression was analyzed using the ∆∆CT method, and the CT value of the gene of interest was compared with that of the housekeeping gene GAPDH. Primer sequences used for qPCR were as follows: *GAPDH*, Forward-TGCACCACCAACTGCTTAGC, Reverse-GGCATGGACTGTGGTGATGAG; *SOX2*, Forward-TACAGCATGTCCTACTCGCAG, Reverse-GAGGAAGAGGTAACCACAGGG; *NTRK2*, Forward-TTACGGTTTGTCACCCGACC, Reverse-CCCGGTCCCTAATTCACACC; *BZLF1*, Forward-TACAAGAATCGGGTGGCTTC, Reverse-GCACATCTGCTTCAACAGGA; *EBER1*, Forward-AGGACCTACGCTGCCCTAGA, Reverse-AAAACATGCGGACCACCAGC; ELF3,Forward-CATGACCTACGAGAAGCTGAGC, Reverse-GACTCTGGAGAACCTCTTCCTC.

### Western blot

A pellet containing 2 × 10⁶ cells was lysed in a buffer containing 1:1 RIPA buffer (Sodium chloride 150 mM, Tris-HCl 50 mM, Nonidet-P40 1%, Sodium deoxycholate 0.5%, SDS 0.1%) and 8 M Urea together with cOmplete Protease Inhibitor Cocktail (Sigma–Aldrich, 11836145001) for 30 min on ice. The cell lysate was centrifuged at 1000 g for 5 min, then the supernatant was collected and protein concentration was measured using Nanodrop 2000/2000c. 5×loading buffer (Tris-HCl 250 mM, SDS 8%, Bromophenol blue 0.1%, Glycerol 40% v/v, Dithiothreitol 100 mM) was incubated at 42 °C for 15 min. Protein fractionation was carried out using homemade SDS-PAGE gel in the Mini Gel Tank system (Invitrogen), followed by transfer onto an Immun-Blot PVDF Membrane (Bio-Rad) using the Mini Blot Module (Invitrogen). Subsequently, the membrane was blocked with TBST milk (Tris-HCl 20 mM, NaCl 150 mM, Tween-20 detergent: 0.1% (w/v)) for 1 h at room temperature. After washing with TBST, the membrane was incubated with primary antibodies diluted in BSA-TBST (4% BSA) buffer overnight at 4 °C. The primary antibodies used were: anti-TrkB antibody [EPR1294] (Abcam, ab134155), Recombinant Anti-SOX2 antibody [EPR3131] (Abcam, ab92494), Anti-EBV ZEBRA Antibody (BZ1) (Santa Cruz Biotechnology, sc-53904, 1:1000), Phospho-S6 Ribosomal Protein (Ser235/236) (D57.2.2E) XP Rabbit mAb (Cell Signaling, 4858), anti-GAPDH antibody (6C5) (Abcam, ab8245), Involucrin Monoclonal Antibody (SY5) (Thermo Fisher, MA5-11803, 1:1000), Phospho-SMAD2 (Ser465/467) (138D4) Rabbit mAb (Cell Signaling, #3108, 1:1000), Phospho-SMAD3 (Ser423/425) (C25A9) Rabbit mAb (Cell Signaling, #9520, 1:1000), SMAD2 (D43B4) XP® Rabbit Monoclonal mAb (Cell Signaling, #5339, 1:1000), SMAD3 (C67H9) Rabbit mAb (Cell Signaling, #9523, 1:1000), TGF beta Receptor 2/TGFBR2 Antibody (D-2) (Santa Cruz Biotechnology, sc-17799, 1:10,000), and Anti-β-Actin antibody produced in mouse (Sigma–Aldrich, clone AC-74, 1:100,000). Following further washing with TBST, the membrane was incubated with secondary antibodies conjugated with horseradish peroxidase (HRP) (Goat Anti-Rabbit IgG H&L (HRP) (Abcam, ab7090), Goat Anti-Mouse IgG H&L (HRP) (Abcam, ab205719)). Finally, the membrane was exposed to Clarity Western ECL Substrate (Bio-Rad) to visualize the signal, which was captured using the ChemiDoc Imaging System (Bio-Rad).

### Spheroids forming assay and immunofluorescence

Cells were sorted using the BD Influx Cell Sorter and seeded into Costar 6-well Clear Flat Bottom Ultra-Low Attachment Multiple Well Plates (CORNING) at a density of 13000 cells per well. The cells were cultured in normal medium supplemented with gentamicin (10 mg/mL) (Gibco 15710064) for one week before harvest. Spheroids were collected by centrifugation in 15 ml tubes at 200 g for 5 min. After removal of the excess supernatant, approximately 100 μl of medium was left to resuspend the pellet. The suspension was then transferred to a 96 well tissue culture plate for counting and imaging.

Immunofluorescence staining was performed as previously described [33]. Briefly, spheroids were fixed with paraformaldehyde (PFA) at 4 °C for 45 min and blocked using PBS containing 0.1% Triton-100 and 0.2% BSA. Processed spheroids were subsequently incubated with primary antibodies (TrkB Antibody (F-1) (Santa Cruz, sc-377218), Recombinant Anti-SOX2 antibody [EPR3131]) in staining buffer overnight at 4°C with gentle shaking. Following primary antibody incubation, spheroids were washed with organoid washing buffer and incubated with secondary antibodies (Goat Anti-Mouse IgG H&L (Alexa Fluor 647), Goat Anti-Rabbit IgG H&L (Alexa Fluor 488)) for 1 h at room temperature. After immunostaining and washing, the spheroids were cleared with fructose-glycerol clearing solution at room temperature for 20 min. Subsequently, the spheroids were mounted with DAPI mounting buffer (Abcam, ab104139) on slides and imaged using a Leica SP8 confocal microscope.

### Cohorts of patients

Written patient consents were obtained from all patients in this study according to institutional clinical research approval. The study protocol (2013.229) was approved by the Joint Chinese University of Hong Kong-New Territories East Cluster Clinical Research Ethics Committee at the Chinese University of Hong Kong, Hong Kong SAR.

### Histologic analysis

Tissue samples were obtained from formalin-fixed paraffin-embedded (FFPE) tumor tissues. Slides (5 ± 1 μm) were prepared by deparaffinizing them through xylene and ethanol washes. Hydrogen peroxide was added to inhibit endogenous peroxidase activity. Antigen retrieval was performed by boiling the slides in an antigen retrieval solution. Endogenous peroxidase activity was further blocked with 3% hydrogen peroxide. Blocking was carried out in 10% normal serum (goat and donkey, Abcam, ab7475, ab7481) with 1% BSA in PBS-T buffer (containing 0.05% Triton X-100). The sections were then incubated with primary antibodies (TrkB antibody, CST4607, diluted at 1:100; SOX2 antibody, CST3579, diluted at 1:200) in a moist chamber. After primary antibody incubation, the sections were incubated with a horseradish peroxidase-labeled secondary antibody. Immunoreactivity was visualized by developing with 3,3′-diaminobenzidine. Finally, the sections were counterstained with hematoxylin (Sigma) to illustrate nuclear detail in cells.

### RNA-scope and data quantification

For RNA-scope analysis, the 2.5 HD Duplex Reagent Kit, along with RNA-scope Probe-Hs-*SOX2* and RNA-scope Probe-Hs-*NTRK2*-C2, were obtained from Advanced Cell Diagnostics. The experiment followed the protocol outlined in the RNA-scope 2.5 HD Duplex Detection Kit (Green/Red) Quick Guide (For FFPE Tissues).

For tissue samples, slides were prepared from formalin-fixed paraffin-embedded (FFPE) tumor tissues. Ten tissues were selected, categorized based on long or short survival records, and cut into 5 ± 1 μm sections, which were then mounted on Superfrost Plus slides. Prior to RNA-scope analysis, the slides were deparaffinized by washing with xylene followed by ethanol. Hydrogen peroxide was added to inhibit endogenous peroxidase activity, and antigen retrieval was performed by boiling the slides in antigen retrieval solution. Following antigen retrieval, the slides were treated with Protease Plus and incubated at 40 °C for 30 min to prepare the sections for the duplex assay. A 1:50 ratio of *NTRK2* probe to *SOX2* probe was prepared, and staining was performed according to the manufacturer’s instructions.

After staining, the slides were imaged under a microscope, with two or three locations on each slide captured for signal quantification. Using Fiji (2.1.0/1.53c) and Python (3.9) with custom code available on GitHub, the red and green signals representing *SOX2* and *NTRK2* expression, respectively, were identified, along with the nucleus from bright-field images. The identified areas were counted, and the signal intensity was quantified based on the pixels of these areas. The signal intensity was then divided by the total area of the nucleus for normalization.

### siRNA knockdown and shRNA knockdown

To perform siRNA knockdown, NPC43 and C17 cells were seeded in a 6-well plate. Upon reaching 60% confluence, the culture medium was refreshed, and the cells were transfected with siRNAs using Lipofectamine RNAiMAX transfection reagent (Invitrogen, 13778075). The specific siRNA sequences used were as follows: *SOX2*: CTGCAGTACAACTCCATGATT; *NTRK2*: ACCACGAACAGAAGTAAT; Negative control (NC): GTCGTCCATTTCCGATTTATT.

Lentiviral vectors were used for shRNA knockdown and overexpression, respectively. Human Embryonic Kidney 293 (HEK293) cells were seeded in a 6-well plate at a density of 2 × 10^6^ cells in 3 ml of medium. Prior to cell seeding, plates were pre-coated with poly-D-lysine to enhance cell attachment. shRNA (pS1H1-H1-copGFP) and overexpression (lentiCRISPR v2) vectors were transfected into HEK293 cells along with VSV-G-expressing envelope plasmid (oMD2.G) and pCMVR 8.74 packaging vector using Lipofectamine 3000 (Invitrogen, L3000150). Following transfection, cells were cultured overnight, and on the second day post-transfection, the medium was replenished, and the previous medium was collected to harvest packaged virus. The collected medium was centrifuged to remove cell debris, and the supernatant containing viral particles was added to target cell lines along with filter-sterilized polybrene to facilitate virus transfection. This process was repeated on the third day post-transfection, and the medium in target cell lines was replenished after 24 h. For shRNA-transfected cells, selection was performed based on positive GFP signal, and cells were cultured in the complete medium supplemented with gentamicin for the first 5 passages to prevent contamination. For overexpression cell lines, puromycin selection was carried out to enrich cells with successful transfection. Puromycin selection was terminated once the cells in the control group died. The primer sequences used for shRNA and overexpression are provided below: shRNA-*NTRK2*-forward: GATCCGTCCAAATGTTTAGCTTAGGTTTCAAGAGAACCTAAGCTAAACATTTGGACTTTTTTG; shRNA-*NTRK2*-reverse: AATTCAAAAAAGTCCAAATGTTTAGCTTAGGTTCTCTTGAAACCTAA GCTAAACATTTGGACG; NC-forward: GATCCGACCAGTCACTACATAAGACATTCAAGAGATGTCTTATGTAGTGACTGGTCTTTTTTG; NC-reverse: AATTCAAAAAAGACCAGTCACTACATAAGACATCTCTTGAATGTCTTATGTAGTGACTGGTCG. *SOX2*-OE-forward: TGCTCTAGAATGTACAACATGATGGAGACGGA; *SOX2*-OE-reverse: CGCGGATCCCATGTGTGAGAGGGGCAGTGT.

### Wound healing assay

In the wound healing assay, cells were seeded into 6-well plates and cultured in complete medium until reaching 100% confluency. Upon reaching confluency, trypsin-EDTA was added briefly to the culture to gently weaken the extracellular connections. The cells were then washed twice with complete medium to neutralize trypsin-EDTA. A p200 pipette tip was used to create a uniform scratch across the culture to ensure a consistent width for each scratch. The medium was replenished with a complete medium supplemented with gentamicin to prevent contamination. Subsequently, the plate was immediately taken for imaging to document the initial status of the wound. The plate was maintained under normal culture conditions, and imaging was repeated after 5 days to assess the final migration status of the cells.

### Visualization

Plots in this study were generated using ggplot2, ggpur, scCustomize, and Plot1cell R packages. The ComplexHeatmap package was used to create heatmaps. The final figures were prepared using Inkscape.

### Statistical analysis

Parametric distributions, including RNA-scope data, and spheroid number and size were analyzed using the Student’s *t*-test to quantify differences between groups. Single-cell differential expression analysis, gene expression, and pathway activities were assessed using the Wilcoxon Signed-Rank Test. For analyses involving multiple groups, such as QPCR ∆∆CT results and number of spheroids, the analysis of variance (ANOVA) test was employed to assess differences and determine statistical significance. The composition analysis of single cells was conducted using scCODA, a Bayesian model designed for compositional single-cell data analysis [34]. A false discovery rate (FDR) threshold of 0.05 was employed for the scCODA analysis. FACS proportion data was analyzed using a two-proportion z-test.

## Results

### The identification of a cell group that is non-responsive to lytic induction

**Fig. 1:**
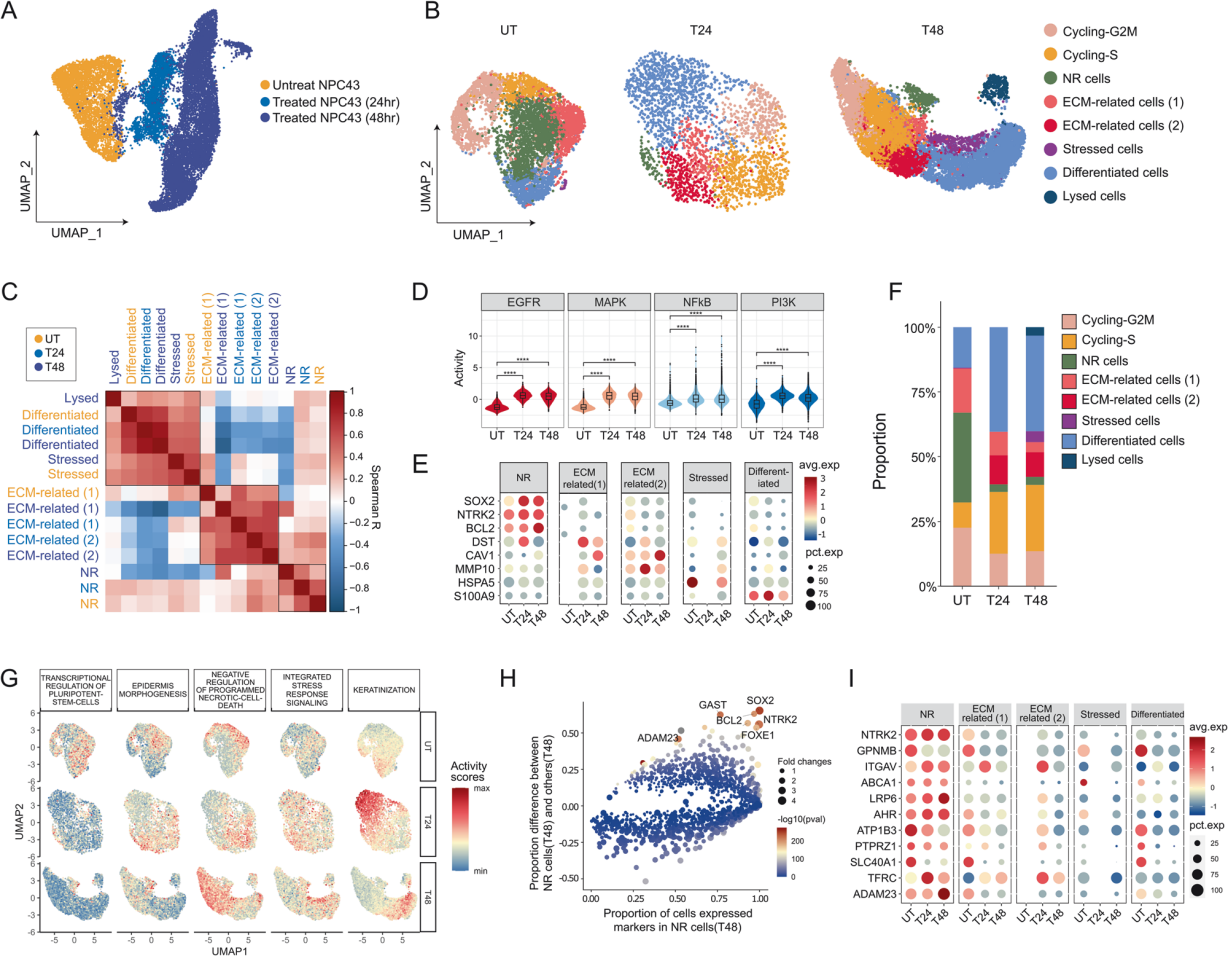
Heterogeneous response of NPC43 cells to lytic induction treatment. **A** UMAP representation of NPC43 cells at three distinct treatment time points, highlighting distinct clustering patterns. **B** UMAP plots illustrating the cellular states at different treatment time points. **C** Heatmap depicting the correlation between pseudo-bulks of different cellular states at various treatment time points, based on the top 1000 variable genes. Heatmap was hierarchically ordered. **D** Violin plots showing the activation levels of the EGFR, MAPK, PI3K, and NF-κB pathways in NPC43 cells. Pathway activity scores were inferred using PROGENy. Wilcoxon signed-rank test was performed. **E** Dot plot illustrating the expression levels of markers associated with different cellular states, demonstrating their conservation across treatment time points. **F** Bar plots showing the percentile distribution of cellular states at different treatment time points. Composition analysis was performed with scCODA, setting FDR to 0.05, and using cycling cells as the reference cell type. The proportions of NR cells, ECM-related cells (2), and Differentiated cells showed credible changes. **G** Scatter plots depicting the activity scores of Gene Ontologies (GOs) differentially activated in cellular states. **H** Scatter plot highlighting the most specific Differentially Expressed genes (DEs) of NR cells at T48. **I** Dot plot showcasing the expression levels of surface markers of NR cells in different cellular states, indicating conservation across different treatment time points.

To investigate the temporal alterations in the transcriptome of NPC43 cells during lytic induction treatment, single-cell RNA sequencing (scRNA-seq) data were generated at distinct time points: untreated (UT), 24 h post-lytic induction treatment (T24), and 48 h post-lytic induction treatment (T48). Uniform Manifold Approximation and Projection (UMAP) was employed for data visualization, revealing a pronounced impact of lytic induction treatment on the transcriptome of NPC43 cells (Fig. 1A and B). Notably, a subgroup of cells from the treated samples tightly clustered with untreated NPC43 cells, indicating a high degree of transcriptomic similarity (Fig. 1A, Supplementary Fig. S1A). To further demonstrate the similarity of these cells, we used hierarchical clustering to reflect the distance between cells, which has been used in our previous work [35]. From this hierarchical clustering heatmap with top variable genes, we further confirmed the similarity between these treated cells and other untreated cells (Supplementary Fig. S1B).

To understand the dynamic changes in the transcriptomes of NPC43 cells, dimension reduction and clustering analyses were performed to categorize NPC43 cells into distinct cellular states at different time points (Fig. 1B, C). Leveraging correlation and integration analyses, cellular states of NPC43 cells before and after lytic induction treatment were defined and corresponded (Fig. 1B, C, and Supplementary Fig. S1C). TPA-based lytic induction treatment resulted in significant global changes in NPC43 cells (Fig. 1A). TPA, a protein kinase C (PKC) activator, stimulates tumor growth in various cancers. To maximize the lytic induction effect on NPC43 cells, the Rho-inhibitor Y-27632 was removed, as it has been shown to suppress TPA-induced EBV lytic replication [36]. To characterize the global pathway changes during lytic induction treatment, we inferred activity scores using PROGENy, focusing on responsive genes within pathways rather than genes playing roles in pathways. This method is believed to more accurately recover pathway perturbations [28]. Our results revealed many pathway perturbations at different treatment time points (Supplementary Fig. S1D). This was expected, as PKC-related activation is a major outcome of lytic induction treatment in NPC43 cells. PKC is a crucial node in various signaling pathways, including MEK/ERK, JNK, p38, and NF-κB [37]. Among these perturbed pathways, EGFR, MAPK, PI3K, and NF-κB showed dramatic changes post-treatment and were consistently increased in replicates (Fig. 1D, and Supplementary Fig. S1D, E). Thus, our longitudinal scRNA-seq data revealed the global transcriptomic changes induced by TPA-based lytic induction.

Next, we sought to characterize the cellular states of single cells. Differential expression analysis and gene set enrichment analysis facilitated the annotation of the biological features of six cellular states (Fig. 1E–G, Supplementary Fig. S2A, B, Supplementary Fig. S3A, and Supplementary Table S1): cells undergoing cell cycles (Cycling-G2M cells and Cycling-S cells), cells expressing high levels of *SOX2* and *NTRK2* which may be non-responsive to lytic induction at the transcriptome level (NR cells), cells expressing numerous matrix metalloproteinases that are related to extracellular matrix (ECM) regulation (ECM-related cells), cells undergoing stress (Stressed cells), cells differentiating towards keratinization (Differentiated cells), and cells undergoing lysis (Lysed cells).

The intriguing population of non-responsive (NR) cells were identified, with proportions decreasing after lytic induction (Fig. 1F). Besides high *SOX2* expression, these cells exhibited heightened activity in Gene Ontology (GO) terms related to stem cell regulation, cell differentiation, and development. Notably, these GO terms were consistently activated post-treatment (Fig. 1G, Supplementary Fig. S3A). Additionally, NR cells may possess anti-apoptotic abilities, as evidenced by high *BCL2* expression (Fig. 1E). *BCL2* is an important apoptosis regulator, deregulated in many cancers, and is currently a promising drug target in clinical trials [38, 39]. ECM-related cells were categorized into two states based on differences in marker gene expression. However, the GO terms enriched in these ECM-related cells were quite similar (Supplementary Fig. S2B, and Supplementary Fig. S3A). Stressed cells, though rare, were distinct, and characterized by GO terms related to the unfolded protein response and glucose starvation (Supplementary Fig. S2B). The proportion of cells from the Differentiated state increased substantially after lytic induction (Fig. 1F). These cells showed numerous detectable EBV genes, indicating a pre-lysed state (Supplementary Fig. S3B, C). Consistent with this observation, cells from Differentiated clusters also exhibited higher inflammatory and viral-budding activity scores, with these pathways’ activity increasing over time (Supplementary Fig. S2C and Supplementary Fig. S3A). Lysed cells expressed markers of initiating EBV lytic cycle, such as *LF3* and *BZLF1* (Supplementary Fig. S2A) [40].

To further elucidate the characteristics of NR cells, a differential expression analysis was conducted to identify markers, particularly surface markers conservatively expressed before and after lytic induction. A list of positive markers for NR cells (T48) was identified, including *SOX2*, *NTRK2*, *FOXE1*, *ADAM23*, *GAST*, *BCL2*, and others (Fig. 1H). Among these markers, a selection of surface proteins robustly expressed before and after lytic induction was chosen, holding promise for the isolation of live cells exhibiting non-responsive properties (Fig. 1I).

The longitudinal design of this study enables the monitoring of dynamic transcriptomic changes in NPC43 cells from untreated states to lytic-induced states. Heterogeneous responses among NPC43 cells were observed, with an increase in the proportion of cells undergoing keratinization and a decrease in cells expressing *SOX2*, termed NR cells, after lytic induction. Importantly, a group of cells with a transcriptome similar to untreated NR cells was identified. This intriguing phenomenon motivates further exploration of the properties of NR cells in NPC.

### The widespread expression of *SOX2* and *NTRK2* in NPC and their prognostic function in the clinic

**Fig. 2:**
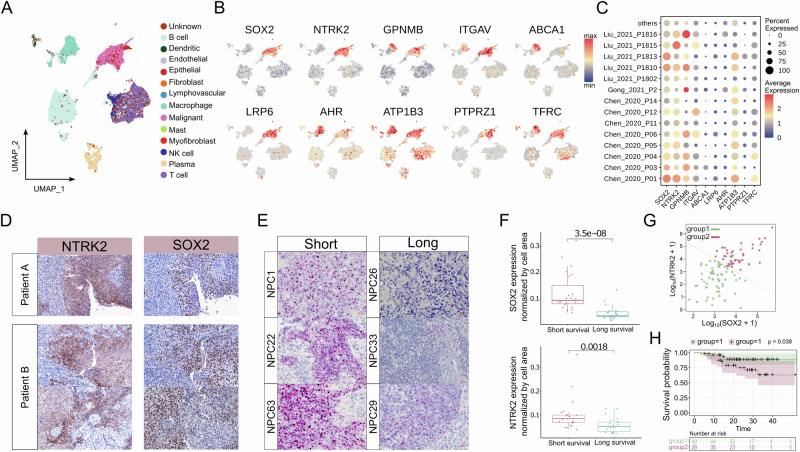
Prevalence of *SOX2*&*NTRK2* in tumor samples from NPC patients. **A** UMAP representation of different cell types in a published NPC dataset. **B** UMAPs displaying the expression levels of *SOX2* and other surface markers in this published NPC dataset. **C** Dot plot illustrating the expression levels of *SOX2* and other surface markers in malignant cells from various published NPC datasets. **D** Immunostaining images demonstrating the detection of SOX2 and TrkB in patient slides. **E** RNA-scope images revealing different abundances of *SOX2* and *NTRK2* in patients with long survival (over 150 weeks) or short survival (less than 50 weeks). *SOX2* is labeled in red, *NTRK2* is labeled in green, and nuclei are labeled in blue. **F** Box plots presenting the quantitative results of RNA-scope; the normalized expression levels of *SOX2* and *NTRK2* in long and short survival patient groups. **G** Scatter plot displaying the correlation between *SOX2* and *NTRK2* using a bulk RNA-seq dataset. Patients were separated into two groups based on *SOX2*/*NTRK2* co-expression. **H** Kaplan–Meier progression-free survival curves for the two groups of patients with high *SOX2*/*NTRK2* co-expression or low co-expression.

Patient-derived cell lines may exhibit gene expression that differs from primary tissue, due to their adaptations to in-vitro culturing and other extrinsic factors. To identify a subset of gene markers from our single-cell dataset with potential clinical significance, we also assessed the expression of surface markers of non-responsive cells in independent datasets of NPC patients. Utilizing a published scRNA-seq dataset comprising 15 tumor samples and one nasopharynx [14], UMAP visualization revealed that *SOX2*, *NTRK2*, *LRP6*, and *PTPRZ1* from our candidate list were predominantly expressed in epithelial and malignant patient cells (Fig. 2A, B). Other surface markers of non-responsive cells identified from our scRNA-seq data displayed less specificity in patient tumor cells. To further validate these markers, we included additional NPC patient samples from other studies to examine the prevalence of these markers in malignant cells (Fig. 2C) [14–18]. Our collective observations indicate the consistent prevalence of *SOX2* and *NTRK2* expression in malignant cells across the various NPC patient cohorts. Immunostaining slides from NPC patients confirmed the existence of the SOX2 and TrkB proteins in this tumor type (Fig. 2D).

*SOX2* is a transcription factor that is highly studied in many biological contexts. It is essential in regulating stem cell differentiation and self-renewal during development; it is one of the four “Yamanaka factors” that can induce pluripotency, and due to its ability to confer stem-like function, it is also associated with CSC phenotypes in many cancers including several lung and colorectal cancer [41–43]. It has previously already been identified as a marker of NPC and potentially plays a role in NPC oncogenesis [44]. *NTRK2* encodes a member of the neurotrophic tyrosine receptor kinase (NTRK) family, presenting three spliced isoforms: one with the intact tyrosine kinase domain (TK) and two with truncated TK, known as T1 and T2 isoforms [45]. Recent clinical and pharmacological studies have focused on *NTRK2* gene fusions [46]. Notably, TrkB.T1 has emerged as the dominant isoform in many cancers [47]. In human glioma, TrkB.T1 has been found to enhance PDGF-driven gliomas in vivo, and in vitro experiments have revealed that TrkB.T1 augments the persistence of PI3K and STAT3 signaling pathways, including pAkt and pS6rp [48]. However, the exploration of TrkB.T1 in NPC or other head and neck cancers has been limited. Given the significance of *SOX2* and *NTRK2* in various studies, we have investigated the interplay between these factors in the context of NPC.

We explored the association between the expression of *SOX2* and *NTRK2* and clinical prognosis by analyzing NPC Formalin-Fixed Paraffin-Embedded (FFPE) sections with RNA-scope (Fig. 2E and Supplementary Fig. S4A). Quantification of *NTRK2* and *SOX2* expression in these RNA-scope data revealed that patients with worse prognoses consistently exhibited higher *SOX2* and *NTRK2* expression (Fig. 2E, F). This finding underscores the potential clinical importance of *SOX2* and *NTRK2*, prompting their utilization in predicting NPC prognosis. Progression-free survival analysis was performed using another NPC tumor cohort with bulk RNA-seq data (Fig. 2G and Supplementary Fig. S4B). Neither *SOX2* nor *NTRK2* alone was effective in predicting NPC prognosis (Supplementary Fig. S4C). However, by categorizing samples into two groups based on co-expression levels: *SOX2*-high/*NTRK2*-high and *SOX2*-low/*NTRK2*-low, we consistently observed results aligning with our previous RNA-scope analysis: patients with higher *SOX2* and *NTRK2* expression had a worse prognosis (Fig. 2G, H).

These findings, based on patient clinical samples, highlight the consistent prevalence of *SOX2* and *NTRK2* in different NPC cohorts. The prognostic analysis suggests the potential important role of *SOX2* and *NTRK2* in NPC tumor progression, prompting further exploration of their function in NP cells. In addition, combining *SOX2* and *NTRK2* could serve as a prognostic marker for evaluating patient survival.

### Non-responsive cells are derived from *SOX2-*high*/NTRK2-*high cells

**Fig. 3:**
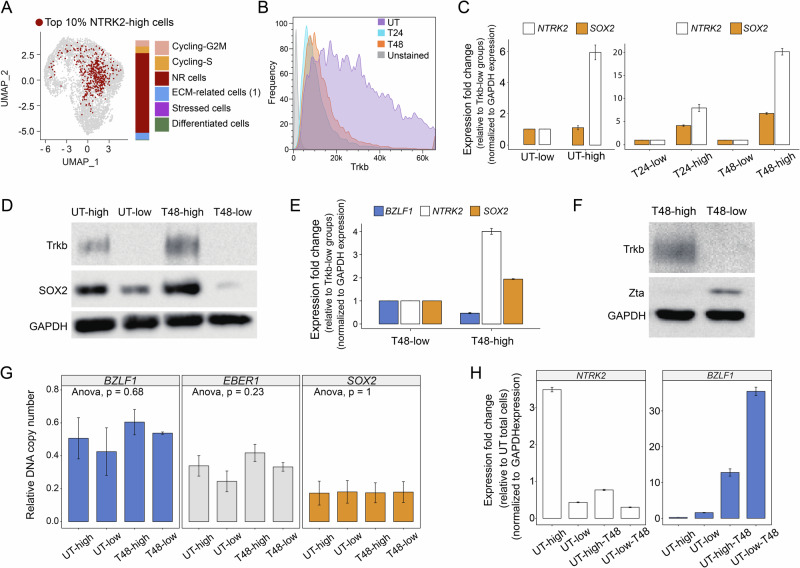
Experimental validation of single-cell RNA-seq results. **A** UMAP representation of the top 10% of cells expressing *NTRK2*, with a bar plot quantifying the proportional distribution of cellular states. **B** FACS detection of TrkB signaling events under different conditions, including an unstained control (unstained), UT, T24, and T48 NPC43 cells. **C** *NTRK2* and *SOX2* expressions determined by qRT-PCR. Cells from different conditions were sorted into NTRK2-high and NTRK2-low groups to extract RNA for qRT-PCR. Significant differences (*p* < 0.05) were observed between T24-high vs. T24-low and T48-high vs. T48-low groups. **D** Western blot showing SOX2 and TrkB abundance in TrkB-high and TrkB-low cells in UT and T48 conditions. GAPDH was used as a loading control. **E** qRT-PCR analysis of NTRK2, SOX2, and BZLF1 expression in FACS-sorted TrkB-high and TrkB-low populations (T48 condition). Significant differences (*p* < 0.05) were detected between T48-high and T48-low groups. **F** Western blot showing TrkB and Zta abundance in TrkB-high and TrkB-low cells in T48 condition. GAPDH was used as a loading control. **G** Relative copy numbers of *BZLF1*, *EBER1* (2 EBV genes), and *SOX2* determined by qPCR. Cells from different conditions were sorted into TrkB-high and TrkB-low groups to extract DNA for qPCR. **H** Expressions of *NTRK2* and *BZLF1* determined by qRT-PCR. Cells were sorted into TrkB-high and TrkB-low groups first (UT-high and UT-low). Then, sorted cells underwent lytic induction treatment for 48 h (UT-high-T48 and UT-low-T48). Significant differences (*p* < 0.05) were observed between UT-high vs. UT-low and UT-high-T48 vs. UT-low-T48 groups.

The preceding analysis underscored the potential importance of *SOX2* and *NTRK2* expression in NPC. Subsequently, to validate results from scRNA-seq at protein level, we examined the presence of SOX2 and TrkB through Western blot analysis in four different cell lines. As anticipated, NPC43 and C17 (EBV+NPC cell lines) exhibited detectable levels of both proteins, while normal nasopharyngeal cell lines NP460 and NP69 lacked endogenous SOX2 and TrkB proteins (Supplementary Fig. S5A). Notably, only the TrkB-T1 isoform, a truncated form of TrkB encoded by the *NTRK2* gene with a size of 93 KDa, was detected in NPC cell lines. This specific *NTRK2* isoform emerged as the dominant transcript variant in several NPC models, as corroborated by bulk RNA-seq data, whereas the full-length isoform, TrkB-FL, was rarely detected (Supplementary Fig. S5B). Since TrkB is a surface protein, we also verified its expression using live cell staining with immunofluorescence and FACS analysis (Supplementary Fig. S5C, D). The top 10% of *NTRK2*-high cells were confirmed to largely correspond to NR cells in scRNA-seq data (Fig. 3A), signifying *NTRK2* as a useful marker for isolating NR cells in NPC43.

Subsequently, we assessed the dynamic changes of TrkB during lytic induction treatment using FACS analysis (Fig. 3B). The population of cells with high TrkB significantly decreased upon lytic induction treatment, consistent with our scRNA-seq data (Fig. 1C). By sorting NPC43 cells into TrkB-high (top 10%) and TrkB-low (bottom 10%) groups before and after treatment, we validated the observed phenomenon that was seen in the scRNA-seq dataset. Both quantitative PCR (qPCR) and Western blot analyses demonstrated a decrease in *SOX2* and *NTRK2* at both the RNA and protein levels (Fig. 3C, D), and notably, *SOX2* and *NTRK2* were consistently co-expressed in a subset of cells.

From scRNA-seq results, NR and ECM-related cells are less differentiated and express significantly fewer EBV genes even after 48 h of lytic induction. To further understand why these cells are non-responsive under lytic activation, we examined the EBV gene expression in *SOX2*-high/*NTRK2*-high cells. Using *BZLF1*, the immediate-early gene initiating the EBV lytic cycle [40], as a marker, we observed lower *BZLF1* expression in TrkB-high cells compared to TrkB-low cells after 48 h of lytic induction treatment, indicating suppressed activation of the lytic cycle in these cells (Fig. 3E, F). Importantly, as determined by DNA qPCR, the reduced *BZLF1* expression in NR cells (TrkB-high) was not attributed to EBV loss but rather other cellular regulations (Fig. 3G).

To investigate the origin of NR cells, we performed integration of the scRNA-seq data and found similar gene expression profiles between a subgroup of untreated cells (untreated NR cells and NR cells at 48 h), indicating that the latter may be derived from the former longitudinally (Fig. 1D, Supplementary Fig. S1C). Trajectory analysis using Partition-based Graph Abstraction (PAGA) revealed connections between NR cells after treatment and those from UT, supporting the hypothesis that NR cells after lytic induction originate from NR cells in UT (Supplementary Fig. S5E, F). To validate findings from scRNA-seq analysis, we sorted untreated cells into two groups, TrkB-high and TrkB-low, and subjected them to lytic induction. Following treatment, qPCR analysis of *NTRK2* and *BZLF1* expression showed that TrkB-high cells maintained higher *NTRK2* expression and exhibited diminished *BZLF1* expression, indicating increased resistance to lytic induction (Fig. 3H). Collectively, these results from scRNA-seq data analysis and induction experiment with sorted cells suggest that *NTRK2*-high cells within untreated NR cells are likely the origin of NR cells observed at T24 and T48.

In summary, TrkB-T1 has been identified as a dominant isoform and cell surface marker in NPC samples. Combining scRNA-seq data and treatment experiments, our results support the hypothesis that non-responsive cells originate from NR cells with *SOX2*-high/*NTRK2*-high expression, which can be enriched by sorting TrkB-high cells. These cells still carry the EBV genome but can resist EBV lytic induction in the presence of lytic induction treatment.

### Lytic induction non-responsive NPC43 cells have CSC-like characteristics

**Fig. 4:**
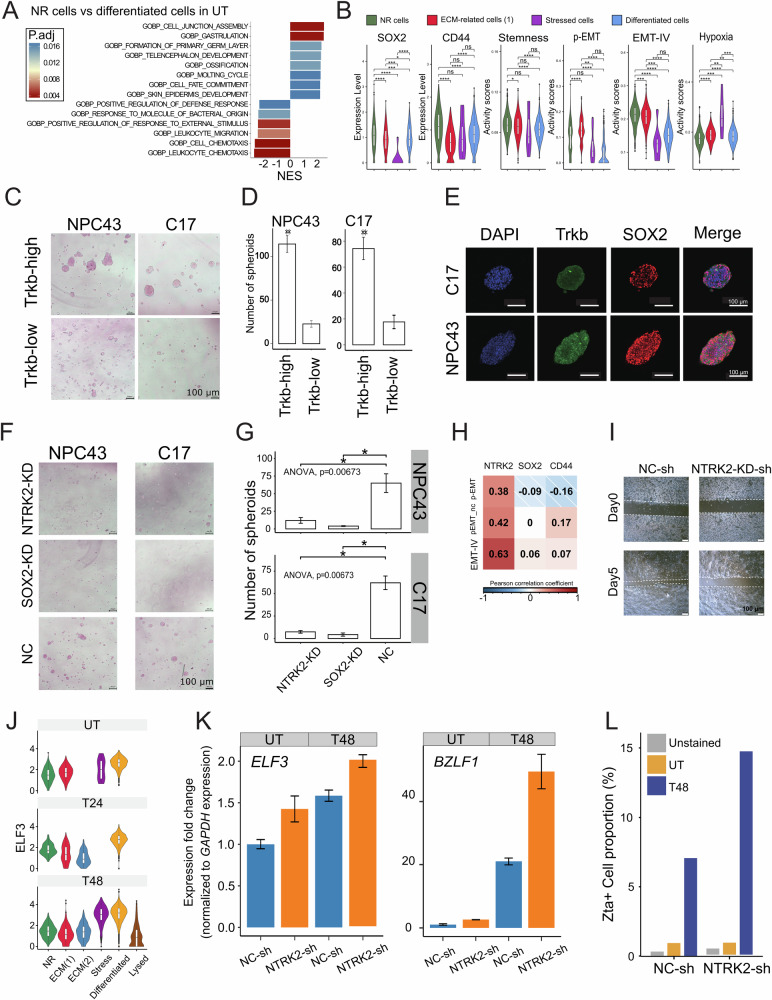
Characterization of NR Cells and the function of *SOX2* and *NTRK2.* **A** GSEA results from DEs between NR cells and Keratinized cells in the UT dataset. **B** Violin plots showing the expression or activity score of genes (*SOX2* and *CD44*) and gene sets (Stemness, p-EMT, EMV-IV, and Hypoxia). Wilcoxon signed-rank test was performed. **C** Images showing the size of tumorspheres. Cells with different TrkB abundance were seeded for this tumorsphere-forming assay. **D** Bar plots showing the numbers of tumorspheres (size over 10 µm). Cells with different TrkB abundance were seeded for this tumorsphere-forming assay. **E** Fluorescent immunostaining of tumorspheres showing the existence of the SOX2 and TrkB. **F** Images showing the size of tumorspheres. TrkB-high NPC43 or C17 cells undergo *SOX2* or *NTRK2* knockdown before seeding for the tumorsphere-forming assay. **G** Bar plots showing the numbers of tumorspheres (size over 10 µm). TrkB-high NPC43 or C17 cells undergo *SOX2* or *NTRK2* knockdown before seeding for tumorsphere-forming assay. **H** Heatmap showing the correlation of *SOX2*, *CD44*, and *NTRK2* with EMT-related gene sets. Three gene sets defined from three different studies consistently show higher correlation with *NTRK2*. **I** Images showing the wound healing assay with different cells. NPC43 cells were knocked down with a shRNA vector. *NTRK2*-KD-sh shows slower migration compared to the control. **J** Violin plots showing *ELF3* expression as a marker of differentiated cells in NPC43. **K** qPCR analysis showing increased expression of *ELF3* and *BZLF1* following *NTRK2* knockdown and lytic induction treatment. Compared to NC-sh of UT, paired *t*-test *p*-values were less than 0.0383 for *ELF3* and 0.00942 for *BZLF1*. **L** FACS analysis of Zta+ cells indicating an increased proportion of cells entering the lytic activation state after *NTRK2* knockdown. A two-proportion z-test showed a *p*-value smaller than 10⁻⁵.

We showed that *SOX2* and *NTRK2* double-positive expression is associated with the outcome of NPC patients, and that they are crucial genes found to be highly expressed in NR cells – a subset of cells exhibiting resistance to lytic induction in NPC43. Next, we explored the biological function of *SOX2* and *NTRK2* double-positive expression in NR cells. We analyzed the scRNA-seq data to identify activated pathways in NR cells, ECM-related cells, and Keratinized cells (Fig. 4A, and Supplementary Fig. S6A), with a focus on the UT dataset. Because in NPC patients, most EBV virus in tumor cells were not activated [49]. Gene Set Enrichment Analysis (GSEA) revealed that NR cells enriched terms related to development (Supplementary Fig. S3A). Genes such as *CD44*, a well-recognized CSCs marker in NPC; gene sets regulating stemness; gene sets involved in epithelial-mesenchymal transition (EMT), and gene sets associated with hypoxia all displayed varying levels of activation in NR cells compared to other cellular states (Fig. 4B), hinting that these cells may possess stronger self-renewal and migration capacity. To examine this hypothesis, we conducted a spheroid-forming assay with NPC43 and C17 cells. We sorted TrkB-high cells, representing *SOX2*-high&*NTRK*-high cells predominantly from NR cells (UT) and performed spheroid-forming assays, demonstrating that TrkB-high cells exhibited higher tumorsphere formation efficiency and larger sphere size (Fig. 4C, D). Immunofluorescence of SOX2 and TrkB in the resulting tumorspheres indicates widespread expression of both markers (Fig. 4E). Subsequently, we assessed the role of *SOX2* and *NTRK2* in tumorsphere formation by performing knockdown experiments (KD) using siRNA. Reduction of either *SOX2* or *NTRK2* led to attenuated spheroid formation in both size and quantity, confirming the essential role of *SOX2* and *NTRK2* in NPC tumorsphere formation (Fig. 4F, G). Western blot analysis confirmed the efficient reduction of SOX2 and TrkB expression after siRNA KD (Supplementary Fig. S6B). Interestingly, *SOX2*-KD also reduced TrkB, suggesting that *SOX2* regulates *NTRK2* expression.

The analysis of gene sets revealed high EMT scores in NR cells (UT). Correlation analysis between genes and EMT scores in the scRNA-seq dataset highlighted a significant correlation between *NTRK2* and EMT-related gene sets (Fig. 4H). To confirm this, we did shRNA-mediated knockdown of *NTRK2* to assess cell migration ability; indeed, reduced *NTRK2* expression weakened cell migration ability in wound healing assays (Fig. 4I, and Supplementary Fig. S6C). Due to the important role of *SOX2* in maintaining stem cell survival, attempts to knockdown *SOX2* to assess its effect on cell migration ability were unsuccessful, because substantial perturbation in *SOX2* expression resulted in massive cell death and cessation of growth in this cell line. This observation is in line with other studies describing *SOX2* as being required for tumor cell survival [23–26]. Moreover, we observed that TrkB-high cells have high repopulation potential, and could give rise to NPC cells with the entire range of TrkB expression levels: Cell populations after 5 days of culture derived from sorted TrkB-high cells largely recapitulate the original broad TrkB expression distribution, while low TrkB expression cells show limited ability to give rise to TrkB-high cells (Supplementary Fig. S6D).

Our previous experiments showed that *NTRK2*-high cells display greater resistance to lytic induction (Fig. 3H). Additionally, our scRNA-seq data revealed that differentiated cells carry higher levels of EBV genes and may represent a prelytic cellular state. Based on these findings, we hypothesized that *NTRK2*/*SOX2* might regulate cellular state transitions, thereby influencing the efficiency of lytic induction.

To test this, we used *NTRK2*-sh cells and examined *ELF3* expression, a marker of differentiated cellular states, using qPCR before and after lytic induction (Fig. 4J, K). We observed a significant increase in *ELF3* expression, indicating an elevated proportion of differentiated cells. Notably, *NTRK2*-sh cells showed a dramatic enhancement in lytic activation, as evidenced by increased *BZLF1* and Zta levels (Fig. 4K, L, Supplementary Fig. S6E). These results support our hypothesis. Furthermore, using an alternative TGF-β method to induce differentiation, we observed similar trends, reinforcing the link between differentiation and lytic resistance (Supplementary Fig. S6F, G).

In conclusion, we demonstrated the significant impact of *SOX2* and *NTRK2* in spheroid formation, wound healing, and the ability to recapitulate parental culture. We also found that *SOX2* regulates *NTRK2* expression. These findings collectively highlight the stem-like characteristics and EMT potential of NR cells. Additionally, we established a connection between stemness and lytic resistance by perturbing *NTRK2* expression. We propose that *NTRK2* influences NPC cell differentiation, ultimately contributing to resistance to lytic cycle entry.

### The regulation function of *SOX2* and *NTRK2* in NPC

**Fig. 5:**
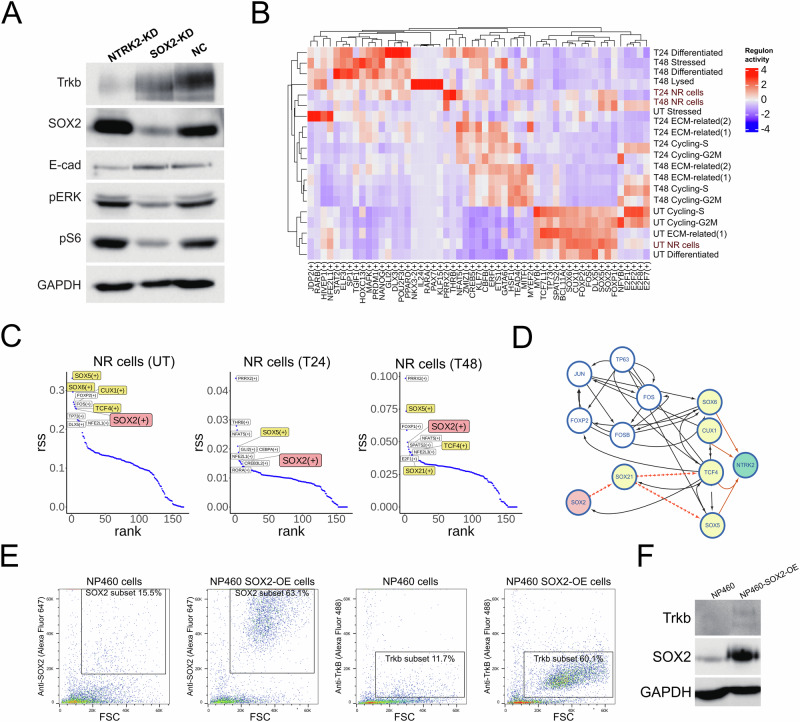
Characterizing the regulation of *SOX2* and *NTRK2.* **A** Western blot showing TrkB, SOX2, E-cad (EMT-related marker), pERK (MAPK-related marker), and pS6 (PI3K-related marker) abundances in NPC43 cells undergoing *SOX2*-KD or *NTRK2*-KD. **B** Heatmap showing the activity of predicted TFs in different conditions. Predicted TFs were inferred from scRNA-seq data with the SENIC algorithm. **C** Rank plots showing the most specified TFs in NR cells at UT, T24, and T48. *SOX2* or *NTRK2*-related TFs were highlighted. **D** Network showing how *SOX2* is connected with *NTRK2*. TFs that are activated in NR cells were visualized in the network. **E** FACS plots showing the detection of SOX2 and TrkB in NP460 cells with *SOX2* overexpression vector. **F** Western blot showing TrkB and SOX2 in NP460 cells with *SOX2* overexpression.

The PI3K/AKT and the MAPK/ERK pathways play pivotal roles in promoting cell cycling, proliferation, and survival in cancers [50, 51], and TrkB.T1 is suggested to be associated with PI3K signaling in glioma [48]. *SOX2* is also implicated in the PI3K/AKT pathway in many cancers, regulating the pathway through a feedforward regulatory manner [52]. Additionally, in embryonic stem cells, the activation of the MAPK/ERK pathway is known to increase the proliferative capacity of SOX2+ cells [53]. Given the importance of PI3K/AKT and MAPK/ERK pathways and their relationship with *SOX2* and *NTRK2*, we investigated whether high *SOX2* and *NTRK2* expression are conferring proliferative and stem-like properties via modulation of the PI3K/AKT and MAPK/ ERK pathways in the induction-non-responsive NPC43 cells. We conducted Western blot analyses to assess the alterations in the PI3K/AKT and MAPK/ERK pathways upon perturbing *SOX2* or *NTRK2*. Our findings indicate that *SOX2* knockdown (*SOX2*-KD) downregulates both PI3K/AKT and MAPK/ERK pathways, while *NTRK2*-KD has no impact on their activity (Fig. 5A). This aligns with the observation that *SOX2*-KD has a more pronounced effect on cell proliferation, leading to the failure of establishing the sh-*SOX2* cell line. Since *NTRK2*-KD does not affect the PI3K pathways, we also examined epithelial-mesenchymal transition (EMT) related markers. E-cadherin (E-cad, *CDH1*), commonly used as an epithelial state marker and thought to be a suppressor of tumor invasion, has been recently implicated in multiple breast cancer models, demonstrating its essential role in detachment, systemic dissemination, and seeding phases of metastasis by limiting reactive oxygen-mediated apoptosis [54]. We observed that *NTRK2*-KD reduces E-cad levels in NPC cells, significantly impacting the formation of tumorspheres (Fig. 5A and Fig. 4F, G). We did not assess N-cadherin (N-cad, *VIM*) as it is rarely expressed in this cell line. Consequently, we posit that *SOX2* is a key upstream regulator for NR cells (UT), governing their growth and stem-like abilities. *SOX2* can also affect *NTRK2* expression, and *NTRK2* is essential for tumor migration, potentially by influencing E-cad levels in a manner similar to breast cancer.

To gain insights into the regulation of *NTRK2* in NPC, we performed gene regulatory network inference (GRN) analysis [55]. Predicted transcription factors (TFs) for different cellular states were identified, revealing the dramatic effect of lytic induction treatment (Fig. 5B). Interestingly, NR cells from T24 and T48 indeed preserved several TF activities when compared with other cellular states, such as *SOX2*, *SOX5*, and *FOXP1* (Fig. 5C). We then investigated the regulatory relationship between *SOX2* and *NTRK2*. We hypothesized that regulators of *NTRK2* would be consistently activated under different conditions (Fig. 5C). Leveraging this information, we discerned possible regulation routes between *SOX2* and *NTRK2*, constructing the regulation network of *NTRK2* (Fig. 5D). From this network, we observed that the possible regulation routes of *SOX2* on *NTRK2* involve *SOX21*, *TCF4*, *SOX5*, *CUX1*, and *SOX6*, whose expression also enriched in NR cells (Supplementary Fig. S7A). *SOX2* itself was not directly identified as a regulator of *NTRK2* in the GRN predictions. Despite this, given the consistent expression of *SOX2* and *NTRK2* across various perturbation conditions in our experiments, we extensively examined many published TF ChIP-seq data, including SOX2 ChIP-seq. Indeed, these ChIP-seq data in other cell types indicated enrichment of *SOX2*, *SOX5*, and *TCF4* in the *NTRK2* gene body, with *SOX2* showing notable enrichment near the promoter region (Supplementary Fig. S7B).

To experimentally validate the regulatory function of *SOX2* on *NTRK2*, we conducted overexpression experiments in NP460 cells, which inherently lack TrkB expression. Our results demonstrated a significant increase in SOX2 protein abundance in NP460-SOX2-OE cells (Fig. 5E, F). Furthermore, TrkB expression was induced by SOX2 overexpression in NP460 cells (Fig. 5E). However, despite this induction, we observed only a slight upregulation of TrkB expression in these cells (Fig. 5F). This modest increase in TrkB expression suggests the intricate regulation of the NTRK2 gene. In NP460, it is conceivable that certain crucial regulators necessary for robust NTRK2 expression may be inactivated, such as potential epigenetic regulatory mechanisms. Additionally, other predicted transcription factors may be required for achieving higher levels of *NTRK2* expression, as they demonstrate binding to regulatory regions near the *NTRK2* gene body in other cell lines. Further investigations into these regulatory factors are warranted to fully elucidate the complex regulation of *NTRK2* in NPC cells.

In summary, we propose that the PI3K/AKT and MAPK/ERK signaling pathways are regulated independently of *NTRK2*, with *SOX2* modulating these pathways. *NTRK2* plays an essential role in tumor migration and tumorsphere formation, with this function potentially related to E-cad levels in NPC43 cells. Additionally, *SOX2* could affect the expression level of *NTRK2*, and even trigger the expression of *NTRK2* from none in normal NP cells. Combining our scRNA-seq data and public CHIP-seq data, we proposed that *SOX2* may not only target *NTRK2* directly but also through the downstream transcription factors of *SOX2*. Moreover, the expression of *NTRK2* may be influenced by gene methylation [56].

### High expression of *NTRK2* and *SOX2* are molecular features of stemness in various NPC models

**Fig. 6:**
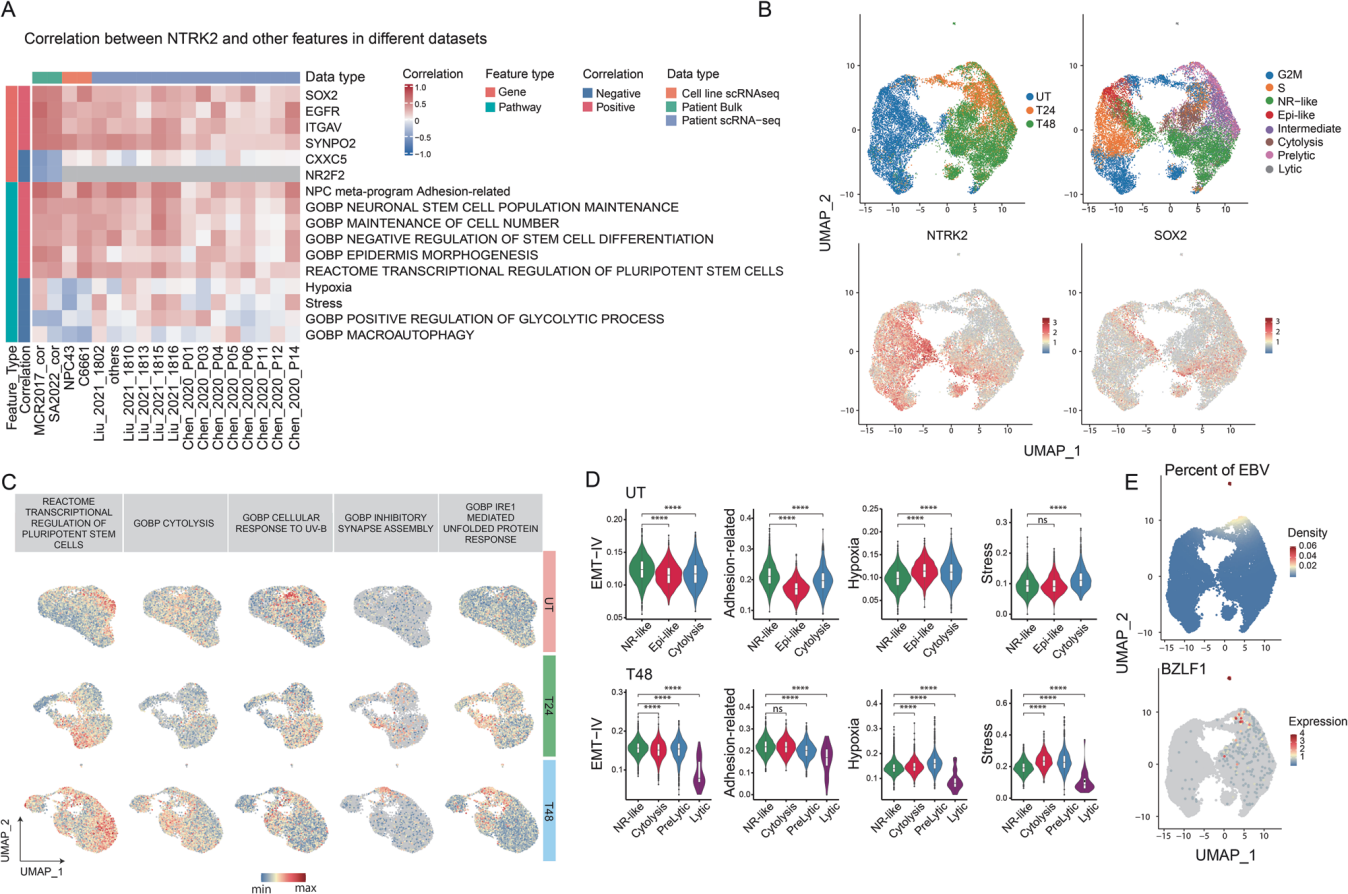
NTRK2 and SOX2 serve as robust features of NR cells across different NPC models. **A** Heatmap displaying the top features correlated with *NTRK2* expression, including genes and pathways. Only features recurrent across multiple datasets are shown in the heatmap. **B** UMAP of integrated scRNA-seq data from C666-1, with cells colored by treatment conditions and cellular states. Expression levels of *NTRK2* and *SOX2* are also visualized on the UMAP. **C** UMAP illustrating representative pathway terms associated with different cellular states. Stemness terms are enriched in NR-like cells; cytolysis and response to UV-B are enriched in cytolysis cells; unfolded protein response and inhibitory synaptic assembly are enriched in Prelytic cells. **D** Violin plot showing scores for EMT, Adhesion-related, hypoxia, and stress pathways. Only UT and T48 samples are included to compare NR-like cells. **E** UMAP shows the density of EBV gene detection and the expression of the lytic activation marker BZLF1.

Through our meta-analysis of NPC patient cell data (Fig. 2C), we validated that *NTRK2* and *SOX2* are highly prevalent in not just NPC43 but also primary NPC tumors. Our phenotypic analysis of NPC43 with *NTRK2* and *SOX2* knock-down highlights the crucial role of these genes in maintaining CSC-related features in NPC43 (Fig. 4). To extend our findings beyond cell lines, we performed a meta-correlation analysis using additional NPC patient datasets. This analysis aimed to further assess the relevance of *NTRK2* and CSC-related features in NPC across a broader range of patient samples (Fig. 6A). First, we found a strong correlation between *NTRK2* and a gene network encompassing *SOX2*, *EGFR*, *ITGAV*, in all NPC models studied, including primary patient samples. Notably, *EGFR* is a well-known oncogene and a promising therapeutic target in multiple cancers, while *ITGAV* has recently been identified as a critical contributor to cancer metastasis [57, 58]. To further elucidate the biological context of *NTRK2*, we investigated its correlation with pathway activity, to capture collective gene expression changes associated with specific biological processes. We curated pathways from diverse sources, including GO, Reactome, pan-cancer gene sets, and NPC-specific gene sets (Supplementary Table S2), and estimated pathway activity scores based on gene expression data. Meta-correlation analysis of *NTRK2* with these pathways revealed a consistent association with stemness-related pathways across all datasets, reinforcing the important role of *NTRK2* in maintaining stem-like properties. This is also concordant with our previous experimental data in NPC43. Furthermore, we observed a strong positive correlation between *NTRK2* and an adhesion-related gene set. This biological process is related to EMT and aligns with our observations from spheroid formation and migration assays in NPC43. Collectively, these findings suggest that high expression of *NTRK2* and *SOX2* constitutes a core molecular program for stemness across diverse NPC models, including patient tumors.

Next, we conducted additional experiments on the EBV-positive NPC cell line C666-1 to further generalize the presence of a core molecular program for stemness in NPC. Similar to NPC43 experiments, C666-1 was treated with a lytic induction agent, and cells were profiled pre-treatment, and at 24 h and 48 h post-treatment using scRNA-seq. Our aim was to determine if a subset of C666-1 cells exhibited lytic induction resistance akin to NPC43 and if these resistant cells also displayed the molecular features of the core NPC stemness program we identified. In NPC43, TPA can easily induce lytic activation through activation of the PKC and its downstream pathways [11]. C666-1, however, is known to have extremely low susceptibility to TPA induction, with lack of EBV late lytic genes expression and being unable to complete the lytic cycle upon TPA treatment [59]. Thus, we induced lytic activation in C666-1 using sodium butyrate (NaB), an HDAC inhibitor, instead of TPA. NaB and TPA have distinct effects on the treated NPC cells. While TPA minimally affects cell growth, NaB-induced HDAC inhibition is highly cytotoxic to malignant cells, causing G1-phase cell cycle arrest and extensive cell death. As such, we expected markedly different responses to induction by these two agents between the two cell lines. In our scRNA-seq analysis of C666-1 induction, we used a similar analytical workflow as for NPC43 (Supplementary Fig. 8A–E). We annotated cells from all conditions (UT, T24, and T48), revealing the following cell states: Non-responsive-like cells (NR-like), Epithelium-like cells (Epi-like), Cytolysis cells (Cytolysis), Intermediate state cells (Intermediate), Prelytic state cells (Prelytic), Lytic cells (Lytic), and Cycling cells (G2/M and S phase). As expected, NaB treatment significantly altered the transcriptome of C666-1 cells (Fig. 6B), and also caused a dramatic decrease in cycling cells (Supplementary Fig. 9A). Another notable discrepancy between C666-1 and NPC43 lies in the Cytolysis and prelytic cell populations. Cytolysis cells are absent in NPC43 but, in C666-1, exhibit activation of UV damage-related pathways, indicating severe DNA damage (Fig. 6C, Supplementary Fig. 9E). Moreover, prelytic cells in C666-1 resemble stressed cells in NPC43, as evidenced by the activation of the unfolded protein response pathway (Fig. 6C). In contrast, the prelytic stage in NPC43 is characterized by differentiated cells.

The majority of treated C666-1 cells showed different responses to lytic induction using NaB compared to TPA-treated NPC43. Nonetheless, non-responsive cells (labeled NR-like) again emerged in C666-1 after treatment, and like the NR cells emerging post-treatment in NPC43, they retained greater transcriptomic similarity to their untreated counterparts compared to other cells (Supplementary Fig. 8C). We further validated that the NR-like cellular state in C666-1 closely resembles the NR state in NPC43 (Supplementary Fig. 9B). Additionally, we confirmed that *NTRK2* and *SOX2* were also highly expressed in C666-1 NR-like cells (Fig. 6B). Most importantly, we found that NR-like cells possess the core molecular program for stemness, as evidenced by consistently higher scores for key gene sets, including EMT and stemness-related pathways (Fig. 6C, D). Furthermore, NR-like cells expanded to become the major cell population at 48 h post-treatment, suggesting they are phenotypically resistant to NaB-induced EBV lytic activation. Indeed, gene expression of EBV and the lytic activation marker *BZLF1* were significantly lower in NR-like cells compared to other cellular states at 48 h (Fig. 6E).

The consistent expression of *NTRK2* and *SOX2* in NR cells across different NPC models and patient samples highlights that NR cells represent a conserved cellular state. Furthermore, the prevalence and functional significance of *NTRK2* and *SOX2* suggest that targeting these factors could serve as a promising therapeutic strategy, particularly when combined with lytic induction therapies.

## Discussion

EBV is a prevalent oncogenic virus found in many cancers, including NPC, lymphoma, and others. Targeting EBV for therapy shows promise; however, similar to HIV, EBV can enter a latency state in some tumor cells, making targeting challenging [60]. There is also the “shock and kill” strategy in NPC, namely lytic therapy, utilizing lytic inducers to force EBV-positive tumor cells into lytic cycles and then the tumor cells were lysed or killed with other drugs. However, the efficiency of lytic inducers is currently low [19]. We hypothesized that the heterogeneous response of NPC tumor cells to lytic inducers might be related to their original cellular states. Therefore, it is crucial to identify, isolate, and characterize the cellular state that can resist the lytic inducer.

In this study, the utilization of newly established NPC cell lines with endogenous EBV infection offered a valuable avenue for investigating the heterogeneity of type III undifferentiated NPC tumor cells upon lytic induction. Specifically, in the NPC43 cell line, TPA treatment and Rho inhibitor withdrawal activate EBV particles, inducing simultaneous changes in both human and EBV gene expression. This model uniquely facilitates the exploration of lytic treatment feasibility for this specific cancer type. Using this model, we investigated the reasons behind the low efficiency of lytic induction, employing scRNA-seq to comprehensively profile changes in the human transcriptome and EBV lytic-related genes during induction treatment. Our findings identified a specific cellular state capable of avoiding entry into lytic cycles.

Through longitudinal experiments, a non-responsive cell population expressing high *SOX2* and high *NTRK2* was identified. To further investigate this cell population, we evaluated the clinical prevalence of several surface markers identified from this group of cells and ultimately selected *NTRK2* for isolating these cells. Importantly, the co-expression of *SOX2* and *NTRK2* was found to be related to patient survival. Isolating cells using *NTRK2* confirmed that NR cells are more resistant to lytic induction treatment, prompting further exploration of their properties. scRNA-seq data suggest that these cells may possess stronger self-renewal capability and exhibit EMT activity. Tumorsphere formation assays and wound healing tests confirmed the essential role of *SOX2* and *NTRK2* in these processes. Using biochemical experiments and GRN analysis, we propose *SOX2* as an upstream regulator of *NTRK2*, targeting the PI3K pathway to control cell growth, while *NTRK2* may play a pivotal role in tumor metastasis through interaction with E-cad. Lastly, we identified *NTRK2* and *SOX2* as key components of the stemness meta-program in NPC using meta-analysis and validated that NR-like cells in C666-1 also exhibit similar features to NR cells in NPC43.

Our study indicates that failure of lytic therapy to eliminate NPC tumor cells could be due to the presence of non-responsive cells, which may contribute to relapse. These non-responsive cells share characteristics with drug-resistant cells, including enhanced self-renewal capacity and metastatic potential. Our meta-analysis across multiple patient datasets revealed a strong association between *NTRK2*, *SOX2*, and stemness-related features, suggesting that *NTRK2* and *SOX2* are likely part of a core program enabling some NPC cells to maintain stemness. The identification of NR-like cells in C666-1 further supports this hypothesis. We also demonstrated that knocking down *NTRK2* or activating other differentiation-related pathways increases sensitivity to lytic induction. These findings highlight that tumor cell stemness is critical for lytic resistance and suggest that targeting stemness-related pathways could improve the efficacy of lytic therapy.

Nevertheless, our work has some limitations. Previous studies have shown that cancer cellular states can transition between one another. One notable aspect we did not consider is the transitions between cellular states, particularly comparing NR cells with ECM-related cells, which exhibit more similarity and may undergo transitions. Investigating ECM-related cells to determine their roles in NPC, particularly regarding metastasis, could be a valuable direction for future research. With regard to the metastatic potential of NR cells, we suggest that *NTRK2* may influence E-cadherin, regulating cell migration and self-assembling. This hypothesis is supported by findings in breast cancer, where E-cadherin was shown to be crucial for colony formation in 3D-Matrigel cultures by inhibiting TGFβ-dependent ROS-mediated apoptosis [54]. Additionally, our meta-analysis revealed that *NTRK2* is strongly associated with *ITGAV*, the other key player in tumor metastasis [57, 58]. It is also plausible that *NTRK2* may serve other functions related to the ligand-based activation process. Previous research has identified the *NTRK2-T1* isoform as dominant in glioma, playing a pivotal role in tumor growth and invasion through BDNF-TrkB regulation [48]. Our results validated the critical role of *NTRK2* in maintaining stemness, which significantly impacts lytic activation. However, further studies are needed to elucidate the detailed mechanisms underlying these processes in the context of NPC.

Our study revealed heterogeneous changes in tumor cells following lytic induction treatment and identified an inverse relationship between lytic activation and tumor cell stemness. Notably, we successfully identified, isolated, and characterized a specific cellular state that exhibits non-responsiveness to lytic induction. This state is marked by high expressions of *SOX2* and a specific isoform of *NTRK2* (NM_001007097). Based on our results, we propose that this particular cellular state may represent drug-resistant abilities, displaying characteristics reminiscent of CSCs or TICs. Furthermore, our meta-analysis suggests that *NTRK2* and *SOX2* are likely part of the core program responsible for maintaining stemness in NPC. Importantly, TrkB, a membrane protein, could serve as a functional marker for CSCs in NPC. Our findings also indicate that stemness is essential for NPC cells to sustain EBV in the latent stage, consistent with the well-established association between the undifferentiated subtype of NPC and EBV infection. These insights emphasize the need for more effective lytic induction drugs. Combining these agents with complementary therapies, such as NTRK2 inhibitors or differentiation activators like retinoic acid, could enhance the efficacy of lytic induction treatments by increasing the proportion of lytic-sensitive cells. This combinatorial approach holds great promise for achieving improved therapeutic outcomes in NPC.

## Supplementary information

supplementary material file

## Data Availability

All sequencing data generated in this study have been deposited in the NCBI’s Gene Expression Omnibus (GEO) repository and are accessible through GEO Series accession number GSE266679. The published data used in this study were retrieved from the Curated Cancer Cell Atlas (https://www.weizmann.ac.il/sites/3CA/), and GEO Series accession number GSE102349, and supplementary of the paper [14–18, 27, 31]. The Transcription Factor ChIP-seq data were checked through Cistrome Data Browser (http://cistrome.org/db/#/) [61]. Data were then loaded onto the UCSC Browser for visualization [62, 63].
