# Supplementary material for: Characterizing resistant cellular states in nasopharyngeal carcinoma during EBV lytic induction
Source: Oncogene. 2025 Mar 25;44(23):1805–19. doi: 10.1038/s41388-025-03341-z (PMC12143974; doi:10.1038/s41388-025-03341-z)
Supplement: Supplementary file 1 — supplementary material file [file 41388_2025_3341_MOESM1_ESM.docx]

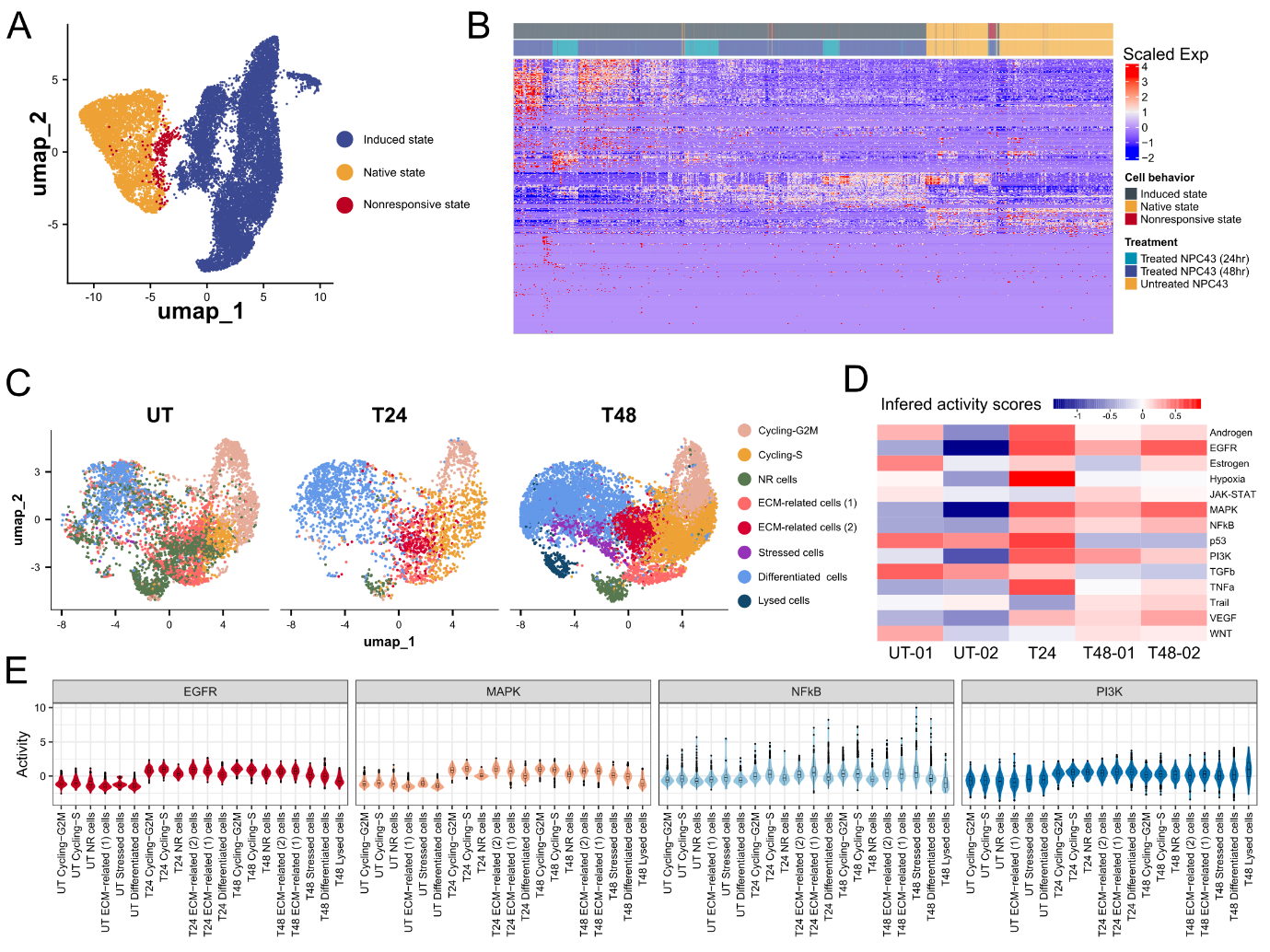


**Supplementary Fig. S1 - Identifying Non-responsive NPC43 Cells to Lytic Induction Treatment. A,** UMAP highlighting the non-responsive cell cluster. **B,** Heatmap showing that hierarchical clustering can also group non-responsive cells with UT cells (using the top 1000 variable genes). **C,** UMAPs showing that after integration, NR cells from UT, T24, and T48 were integrated. Other states were also integrated well. **D,** Heatmap displaying the activation levels of 14 pathways in NPC43 cells before and after lytic induction treatment. Pathway activity scores were inferred using PROGENy. **E,** Violin plots showing the activation levels of the EGFR, MAPK, PI3K, and NF-κB pathways in NPC43 cells at the cellular state level. Pathway activity scores were inferred using PROGENy.

**
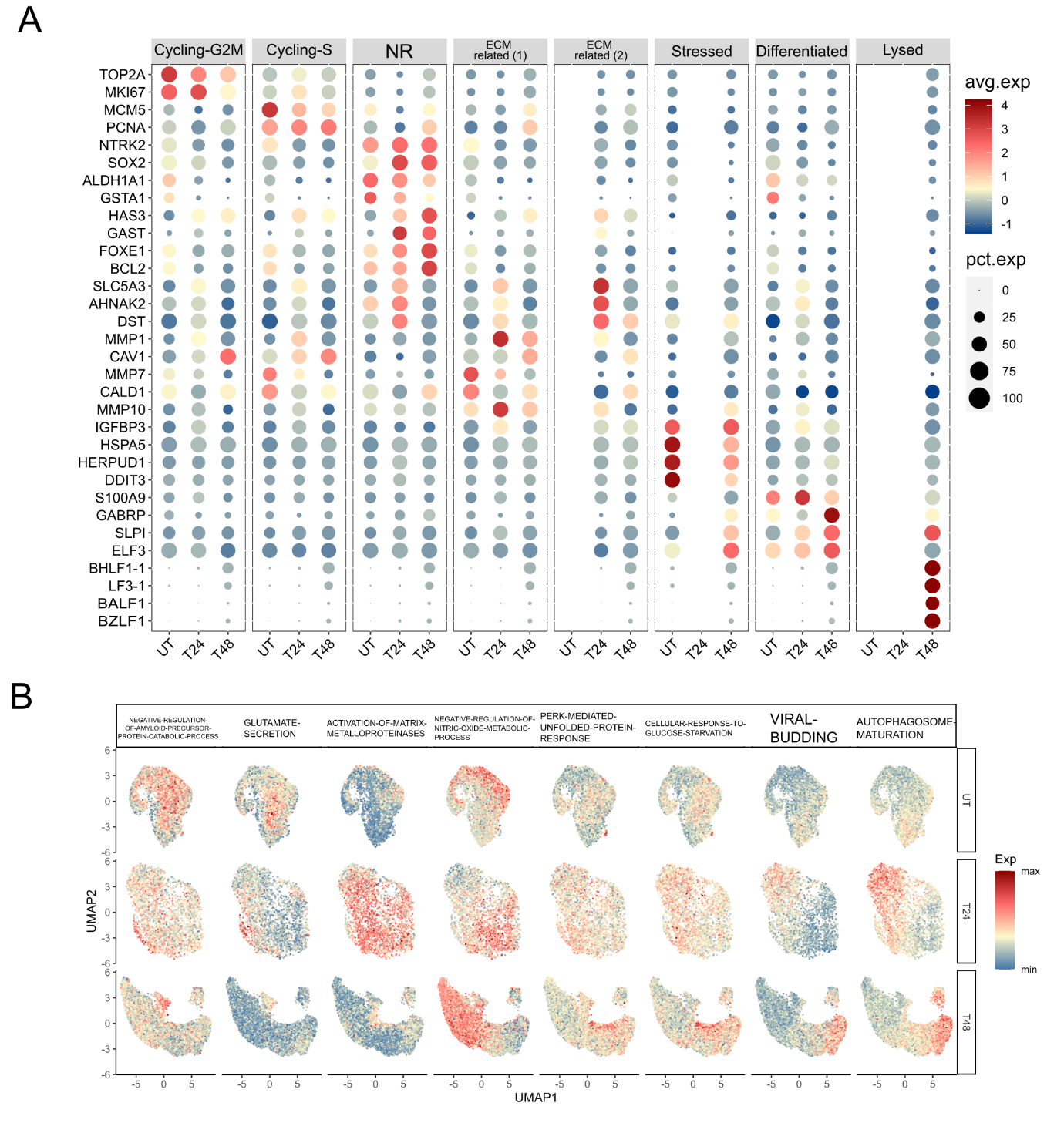
**

**Supplementary Fig. S2 - Markers and Activated GOs in Different States of Cells. A,** Dot plot showing the top 5 markers in different states of cells. **B**. UMAPs showing some significant Gene Ontology (GO) terms that are differentially activated in different states of cells.

**
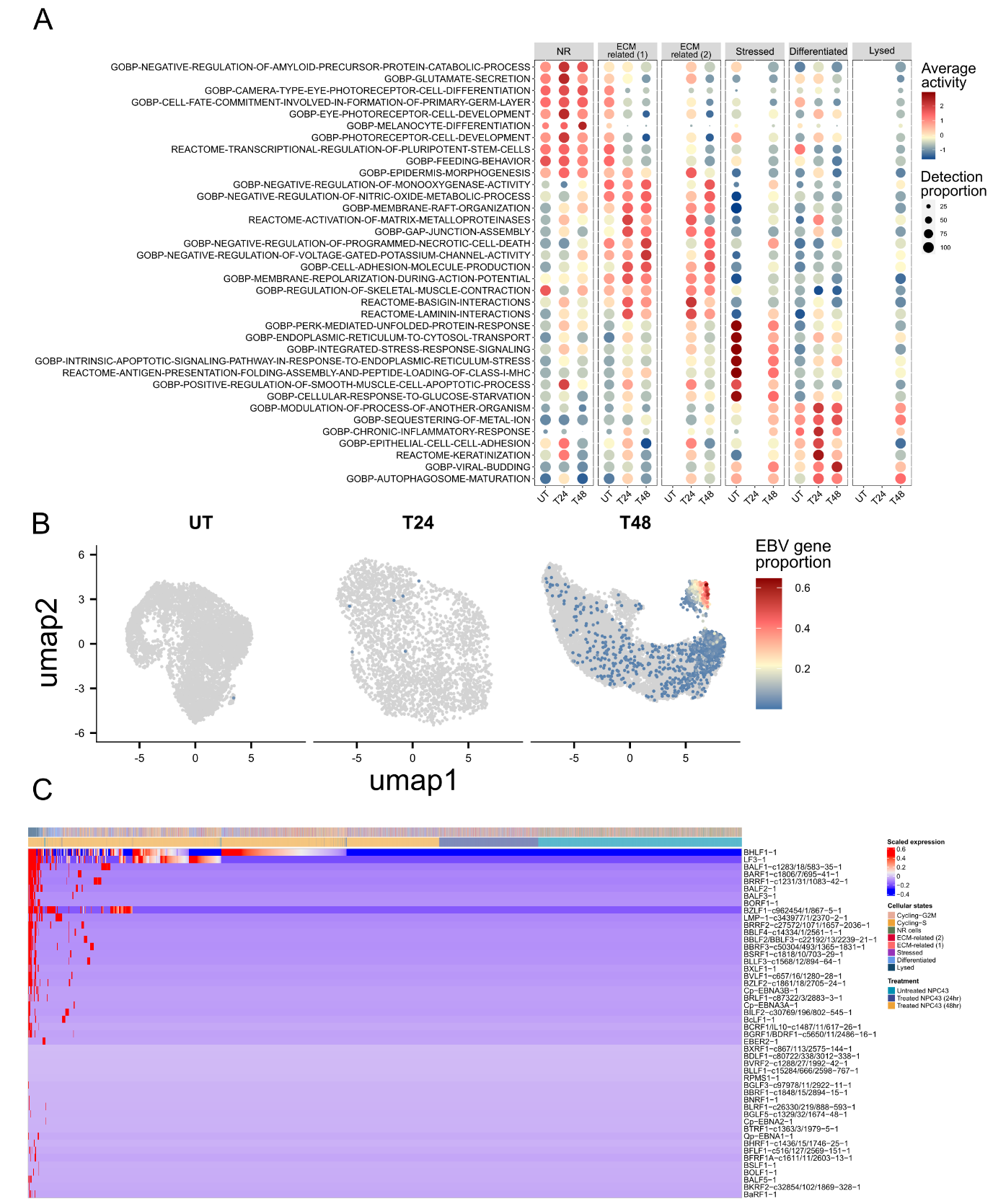
**

**Supplementary Fig. S3 - Activated GOs in Different States of Cells. A,** Dot plot showing the top 10 differentially activated Gene Ontology (GO) terms in different states of cells. **B,** UMAPs showing the proportion of Epstein-Barr virus (EBV) gene reads in cells. **C,** Heatmap showing the detected EBV gene expression in single cells before and after the lytic induction treatment.

| **Annotation names** | **Short names** | **Markers** | **GO features** |
| --- | --- | --- | --- |
| Cycling-G2M cells | Cycling-G2M | *TOP2A; MKI67* | Cell cycles |
| Cycling-S cells | Cycling-S | *MCM5; PCNA* | Cell cycles |
| Non-responsive cells | NR cells / NR | *SOX2; NTRK2; FOXE1; BCL2; ALDH1A1* | Glutamate secretion; Cell development; Cell differentiation |
| ECM-related cells (1) | ECM-related (1) | *MMP10* | Negative regulation of oxide metabolism; Negative regulation of cell death; ECM-related |
| ECM-related cells (2) | ECM-related (2) | *DST* | Negative regulation of oxide metabolism; Negative regulation of cell death; ECM-related |
| Stressed cells | Stressed | *IGFBP3; HSPA5* | Unfolded protein response; Glucose starvation |
| Differentiated cells | Differentiated | *SP100A; ELF3* | Viral budding; Autophagosome maturation; Keratinization |
| Lysed cells | Lysed | *BZLF1* | Viral budding; Autophagosome maturation |

**Supplementary Table S1 - The annotation table of cellular states of NPC43 cells.** Table showing the annotation information of different cellular states of the NPC43 cells.


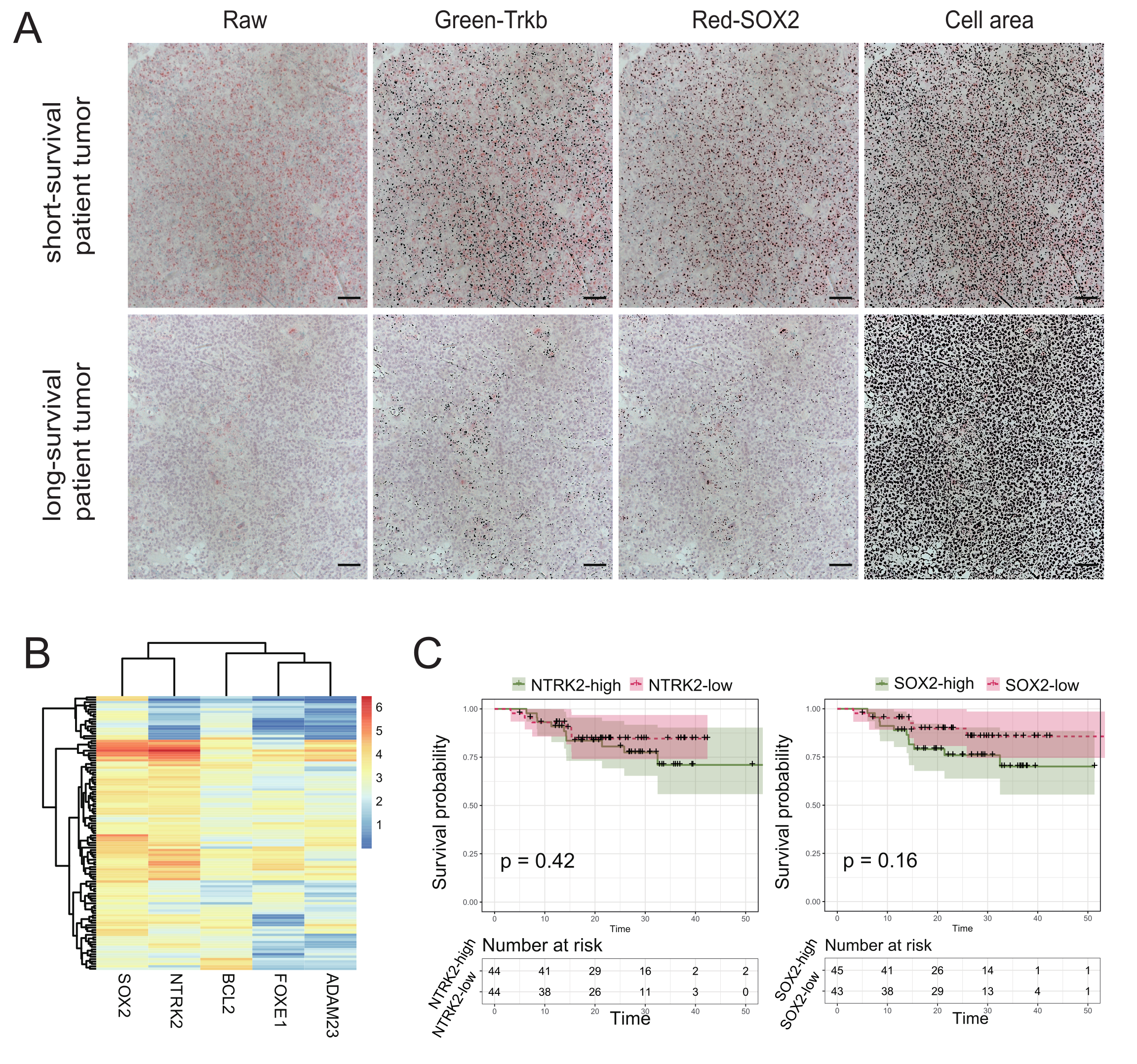


**Supplementary Fig. S4 - The Clinical Significance of *SOX2*&*NTRK2*. A,** An example of RNA-scope data processing. **B,** Heatmap showing marker expression in a bulk RNA-seq dataset. **C,** Kaplan–Meier progression-free survival curves for the two groups of patients based on *SOX2* or *NTRK2* expression.


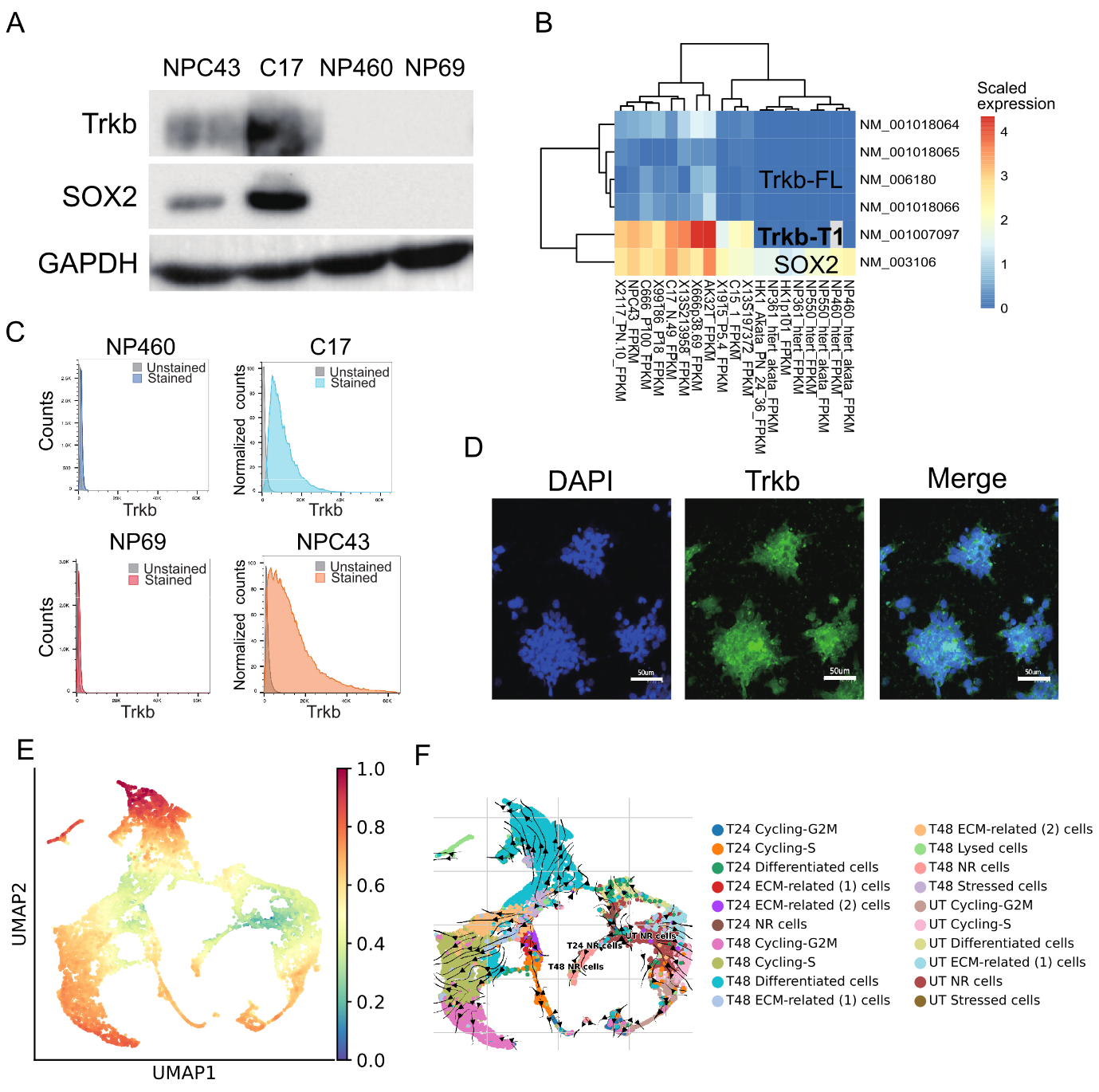


**Supplementary Fig. S5 - Validate the Existence of NTRK2 in NPC or NP cell lines. A,** Western blot illustrating the abundance of TrkB and SOX2 in NPC43, C17, NP460, and NP69 cell lines, with GAPDH as the loading control. **B,** Heatmap presenting the NTRK2 isoforms in NPC cell lines. **C,** FACS detection of TrkB signaling events in NPC43, C17, NP69, and NP460 cells. **D,** Live-cell immunofluorescent staining images depicting TrkB labeling in NPC43 cells. NPC43 live cells were initially stained with TrkB antibody, followed by fixation and subsequent staining with DAPI. **E,** PAGA-initiated UMAP embedding showing a more connected representation of cellular states. Diffusion Pseudotime (DPT) was calculated by selecting untreated (UT) NR cells as the starting point. **F,** PAGA-initiated UMAP displaying a velocity-like plot. Velocity vectors, inferred from DPT, illustrate the trajectory from untreated NR cells to treated NR cells and other untreated cellular states.

**
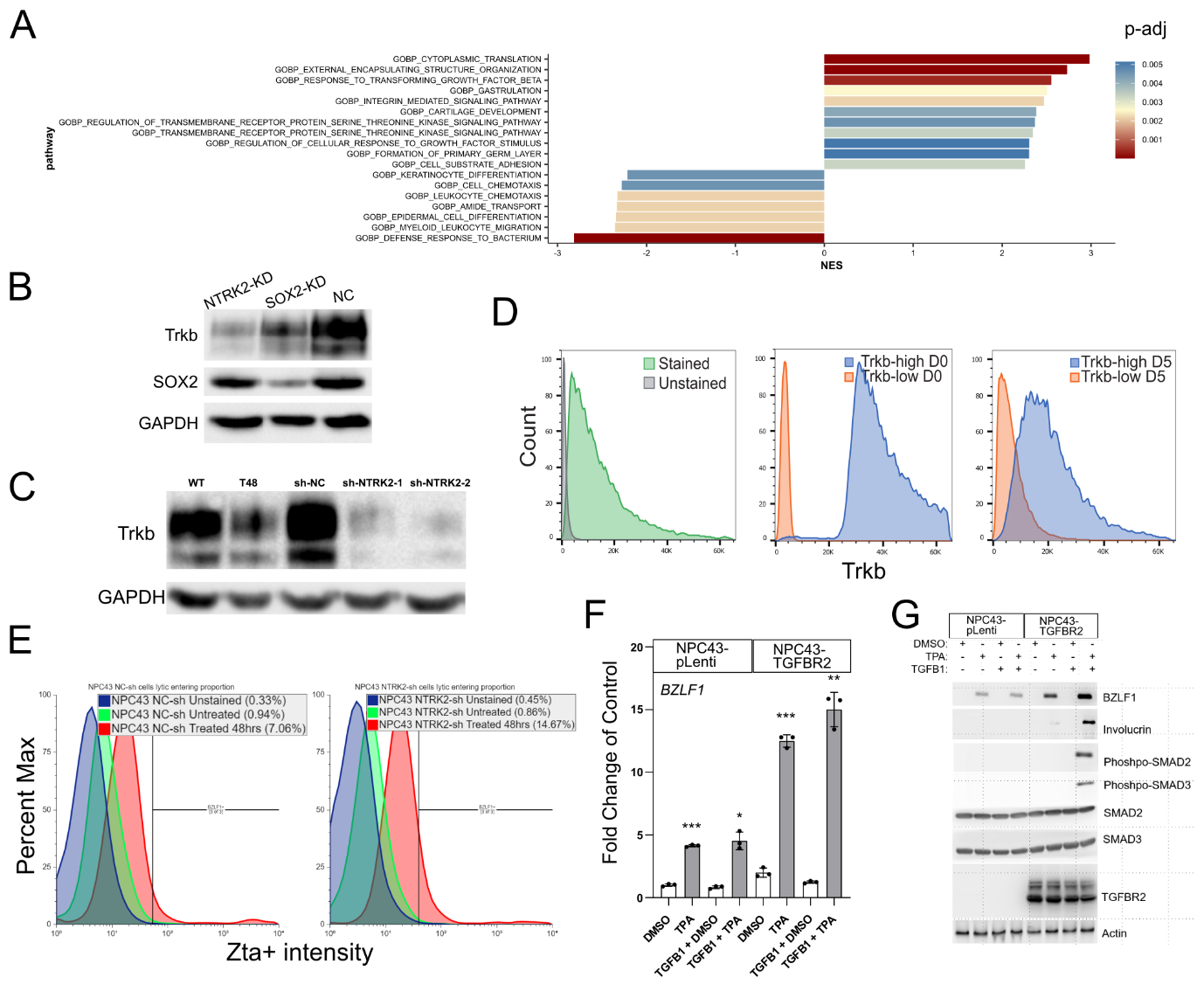
**

**Supplementary Fig. S6 - Characterizing the Function of *NTRK2* and *SOX2* in NR Cells. A,** GSEA result derived from the DEs between ECM-related cells and Keratinized cells in the UT dataset. **B,** Western blot illustrating TrkB and SOX2 abundance in cells with *NTRK2* knockdown (*NTRK2*-KD), *SOX2* knockdown (*SOX2*-KD), and control cells. GAPDH served as the loading control. **C,** Western blot showing TrkB abundance in different NPC43 cell conditions: wild-type, T48 (lytic induction treatment for 48 hours), shRNA control, shRNA targeting *NTRK2* (replicate 1), and shRNA targeting *NTRK2* (replicate 2). **D,** FACS detection of TrkB signaling events in NPC43 cells. TrkB-high and TrkB-low cells were re-analyzed with FACS immediately after sorting and after 5 days of culturing. **E,** Gating plots from FACS analysis showing an increased proportion of Zta+ cells following *NTRK2* knockdown. **F,** qPCR analysis indicating increased expression of *BZLF1* following *TGFBR2* expression and *TGFB1* activation. **G,** Western blot analysis confirming that differentiation was achieved through TGFBR2 expression and TGFB1 activation, with an increased abundance of Zta (*BZLF1*). Members of the differentiation pathway were also examined.


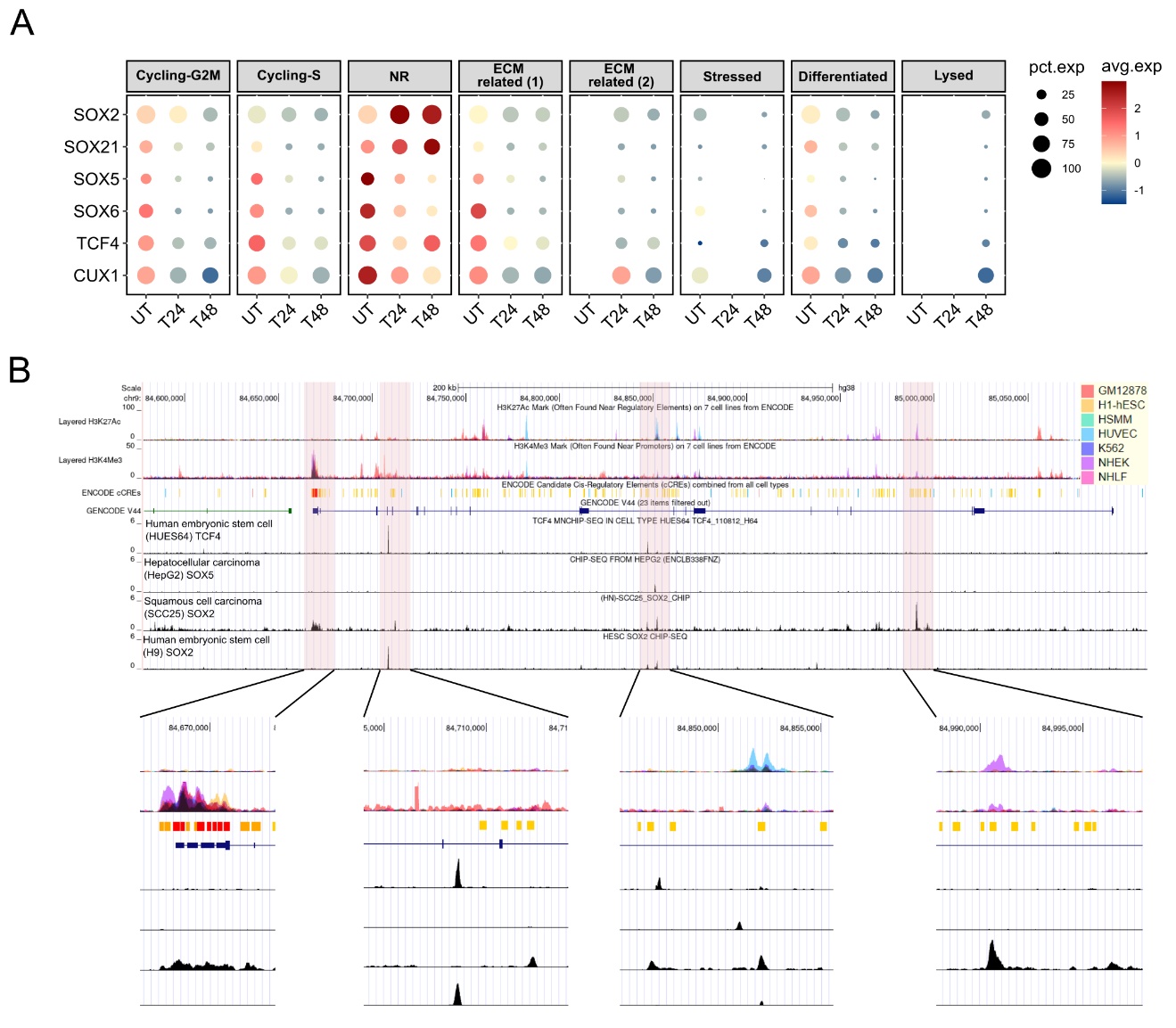


**Supplementary Fig. S7 - Checking the Enrichment of TFs near the NTRK2 gene. A,** Dot plot showing the TFs markers in different states of cells. **B,** *SOX2* ChIP-seq peaks and H3K4Me3 signals colocalize well near the *NTRK2* promotor region. *SOX2*, *SOX5*, and *TCF4* also show enrichment near H3K27ac peaks.


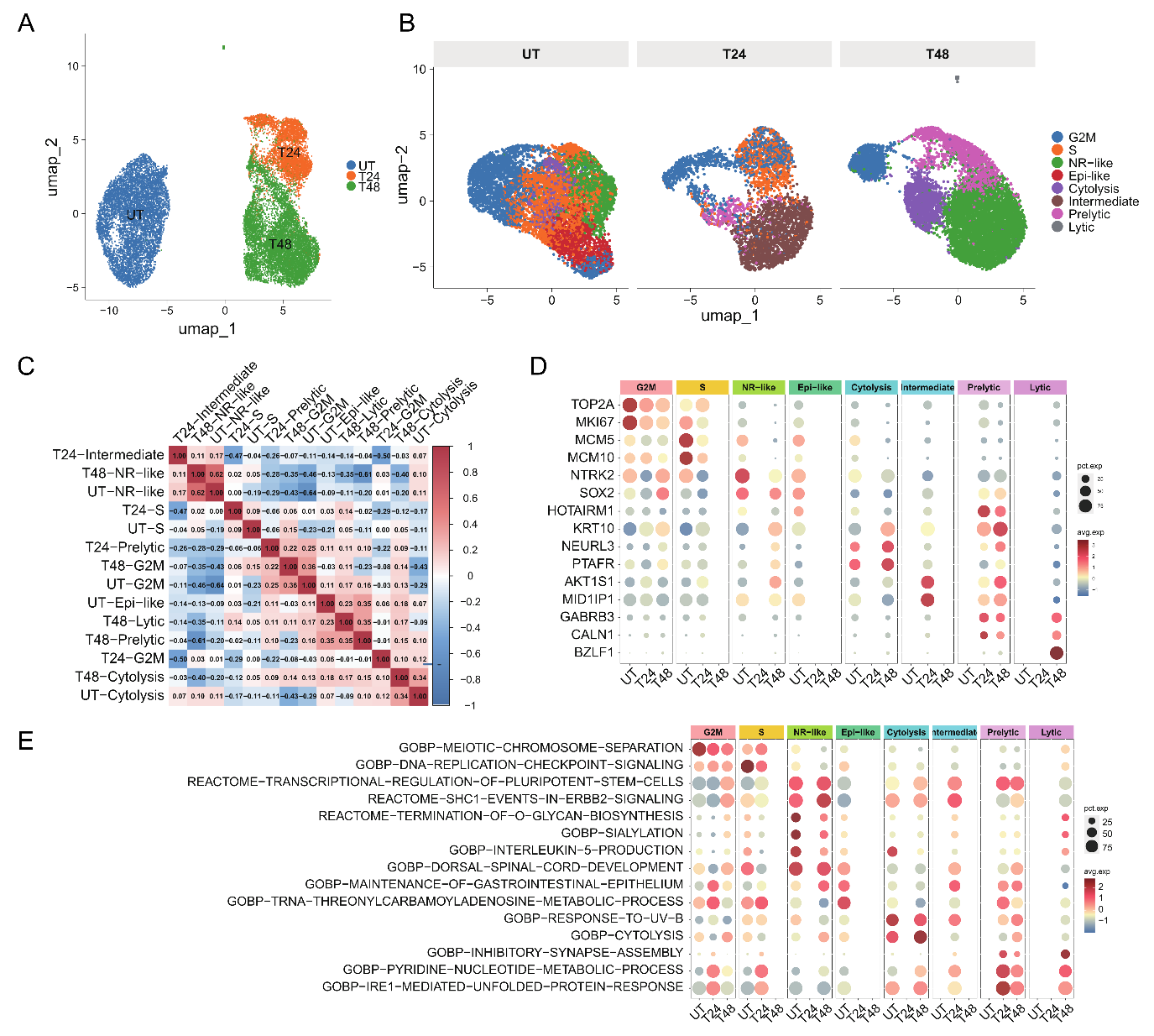


**Supplementary Fig. S8. Annotating Cellular States in C666-1 Cells. A,** UMAP showing the dimensionality reduction results of C666-1 cells, with treatment conditions annotated. **B,** UMAP showing the separated dimensionality reduction results for UT, T24, and T48 samples, with cellular state annotations. **C,** Correlation heatmap showing stronger correlations between NR-like cells from UT and T48 samples, based on commonly identified highly variable genes. **D,** Dot plot displaying key markers identified from scRNA-seq of C666-1 cells across all cellular states. **E,** Dot plot showing the enriched pathway terms for each cellular state.


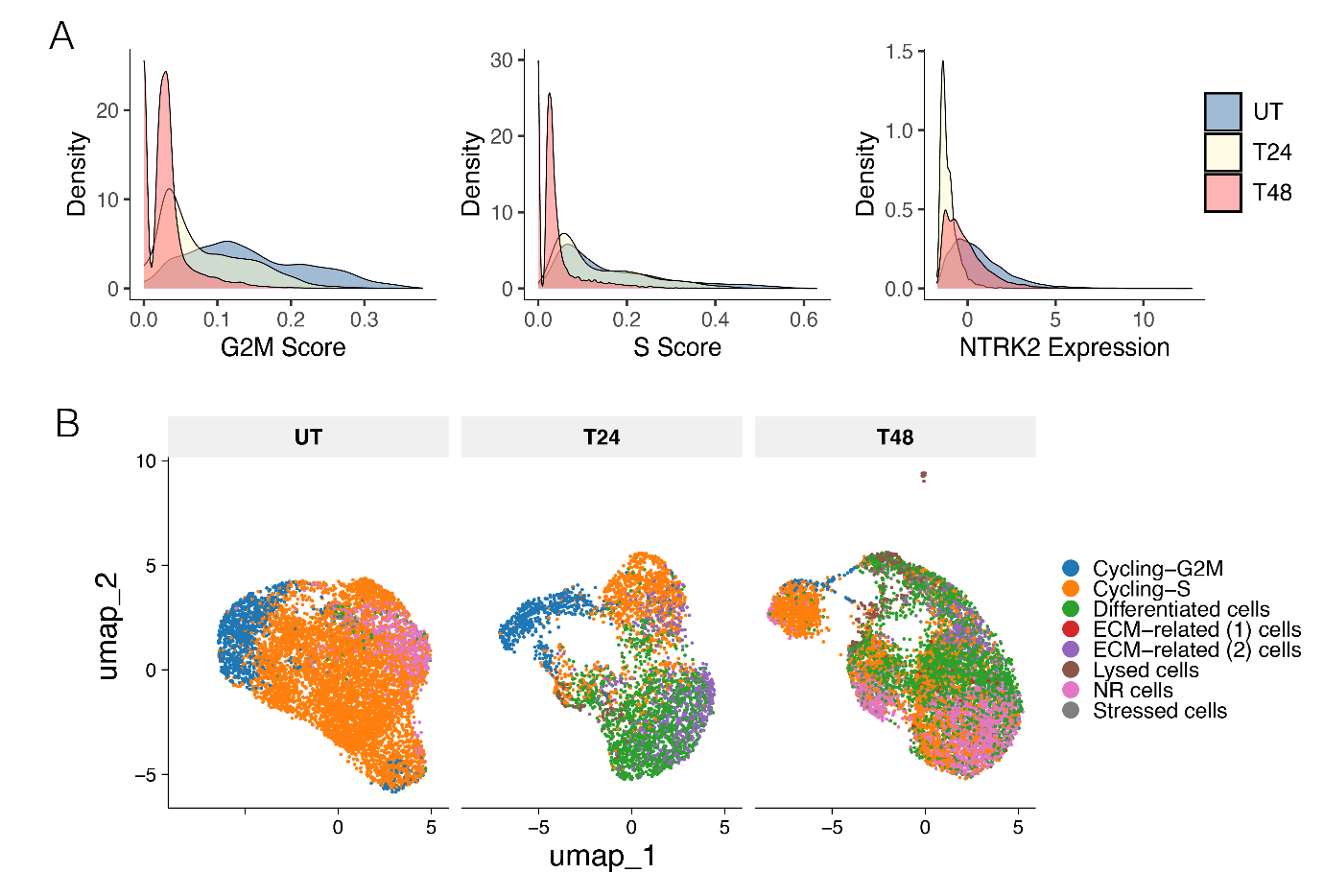
**Supplementary Fig. S9. Annotating Cellular States Using the NPC43 Dataset as a Reference. A,** Density plots showing a dramatic decrease in cycling cells following lytic induction with NaB in C666-1. **B,** UMAPs for each condition (UT, T24, and T48) with mapped cellular state annotations based on the NPC43 dataset.

| **Differentiation-related** | **Cell-cycle-related** | **Immune-related I** | **Immune-related II** | **Adhesion-related** |
| --- | --- | --- | --- | --- |
| GPNMB | HMGB2 | OGFRL1 | LCN2 | DST |
| KRT17 | CDK1 | C6orf141 | C15orf48 | GABPB1-AS1 |
| S100A2 | UBE2T | CCL20 | CFB | MALAT1 |
| KRT6A | MAD2L1 | ICAM1 | SAA1 | ARID1B |
| SFN | TK1 | PRRX1 | AGR2 | FTX |
| TACSTD2 | TYMS | RAI14 | BPIFA1 | HHV4_RPMS1 |
| KRT15 | UBE2C | RELB | BPIFB1 | MIR205HG |
| KRT16 | CENPF | SOCS1 | C3 | NEAT1 |
| NEAT1 | CKS1B | SSTR2 | KRT23 | SYNPO2 |
| SAT1 | NUSAP1 | VCAM1 | LYPD2 | AKAP9 |
| CTSB | TOP2A | VRK2 | MUC1 | ATRX |
| KRT5 | BIRC5 | BCL2A1 | MUC5B | CCDC14 |
| ATF3 | HIST1H4C | BIRC3 | PDZK1IP1 | COL17A1 |
| CLDN4 | PCNA | CIITA | PIGR | DSP |
| DDIT3 | RAD51AP1 | CXCL10 | RARRES1 | GOLGB1 |
| EGR1 | TUBA1B | HLA-DPA1 | RDH10 | IGFBP2 |
| KRT13 | ZWINT | HLA-DPB1 | S100P | KANSL1 |
| NUPR1 | CENPU | HLA-DQA1 | SAA2 | LINC00173 |
| PLAU | CENPW | HLA-DRA | WFDC2 | MIR99AHG |
| PPP1R15A | MKI67 | HLA-DRB1 | CD55 | N4BP2L2 |
| S100A10 | RRM2 | KRT13 | CEACAM6 | PNISR |
| S100A6 | SMC4 | MARCO | CP | POLR2J3 |
| S100A8 | ASPM | MYBPC1 | CXCL1 | PRKDC |
| S100A9 | PCLAF | NPPC | CXCL17 | SYNE2 |
| SOD2 | ASF1B | PNRC1 | IGFBP3 | TNRC6B |
| TNFSF10 | CDC20 | SPIB | KRT7 | ZNF292 |
| ZFP36 | CENPH | SYNPO2 | PRSS23 | FRMD4A |
| BTG2 | CKS2 | PLEK | PSCA | ZKSCAN1 |
| CLEC2B | HELLS | TNFAIP3 | SERPINB3 | KCNQ1OT1 |
| DST | HMGN2 | CLDN3 | SLPI | ATP1A1 |
| DUSP1 | KPNA2 | CCL19 | TFF3 | VCAM1 |
| ELF3 | PRC1 | NTRK2 | UBD | ITGAV |
| FOS | PTTG1 | CAV1 | VMO1 | RIC3 |
| GADD45B | TMEM106C | SNCG | MUC5AC | TFRC |
| HCAR3 | TMPO | TNFAIP2 | ERN2 | CCNL1 |
| HES1 | TPX2 | SPINK5 | ZG16B | HSPA5 |
| ID1 | AURKB | CD74 | PI3 | NFE2L3 |
| IER2 | CCNB1 | GSN | CAPN13 | CD44 |
| KLF6 | CCNB2 | VIM | ADGRF1 | AC020916.1 |
| LAMB3 | CDCA7 | EDARADD | GDF15 | AHNAK |
| NR4A1 | CENPM | CDC42EP4 | ISG15 | GVQW3 |
| RND3 | DHFR | ODC1 | SLC34A2 | PTPRF |
| WFDC2 | H2AFX | GADD45A | TNFAIP2 | HLA-DRB1 |
| SLPI | KIF20B | CALML5 | MDK | BX890604.2 |
| FOSB | MCM3 | TMEM150C | MUC20 | APLP2 |
| NDRG1 | MCM4 | INPP1 | ASS1 | HOOK2 |
| ANXA1 | MCM7 | CALML3 | IFITM1 | PSAP |
| JUN | PBK | MT2A | MX2 | BSG |
| DNAJB1 | KIAA0101 | PTGES | STARD10 | FHOD3 |
| RHOB | STMN1 | LGALS1 | ATF3 | LAMB1 |

**Supplementary Table S2 – NPC-specific gene sets derived from scRNA-seq data of patient tumors.** This table lists five gene sets along with the genes associated with each set.
